# Supplementary material for: Detecting the Critical States of Type 2 Diabetes Mellitus Based on Degree Matrix Network Entropy by Cross-Tissue Analysis
Source: Entropy (Basel). 2022 Sep 5;24(9):1249. doi: 10.3390/e24091249 (PMC9498060; doi:10.3390/e24091249)
Supplement: Supplementary file 1 [file entropy-24-01249-s001.zip › Supplementary Material.pdf]

Supplementary Material: Detecting the Critical States of Type 2 Diabetes Mellitus Based on Degree Matrix Network Entropy by Cross-tissue Analysis

Contents

S1. Comparison of different parameters of CSN 1
S2. The identification of ‘dark genes’ for T2DM with PAAD data 2
S3. Drug target analysis for GSE13268: Rat Adipose Tissue 4
S4. Drug target analysis for GSE13269: Rat Gastrocnemius Muscle Tissue 10
S5. Drug target analysis for GSE13270: Rat Liver: Rat Liver Tissue 16
S6. Enrichment Results and Analysis of GSE13269 (Rat Gastrocnemius Muscle Tissue) and GSE13270 (Rat Liver Tissue) 19
References 24

S1. Comparison of different parameters of CSN

According to the research of Dai et al. [1], the influence of box size and P value in different data sets on clustering effect is tested from the point of view of clustering. It is indicated that the optimum box size is about 0.1, and the optimum P-value is about 0.01 on average, which are set as the default parameters of CSN method. So we choose 0.1*k, and we can adjust the parameters according to the specific needs. From the Figure 1, we can see some different data sets box size is stable and the final estimate of the value not much difference.

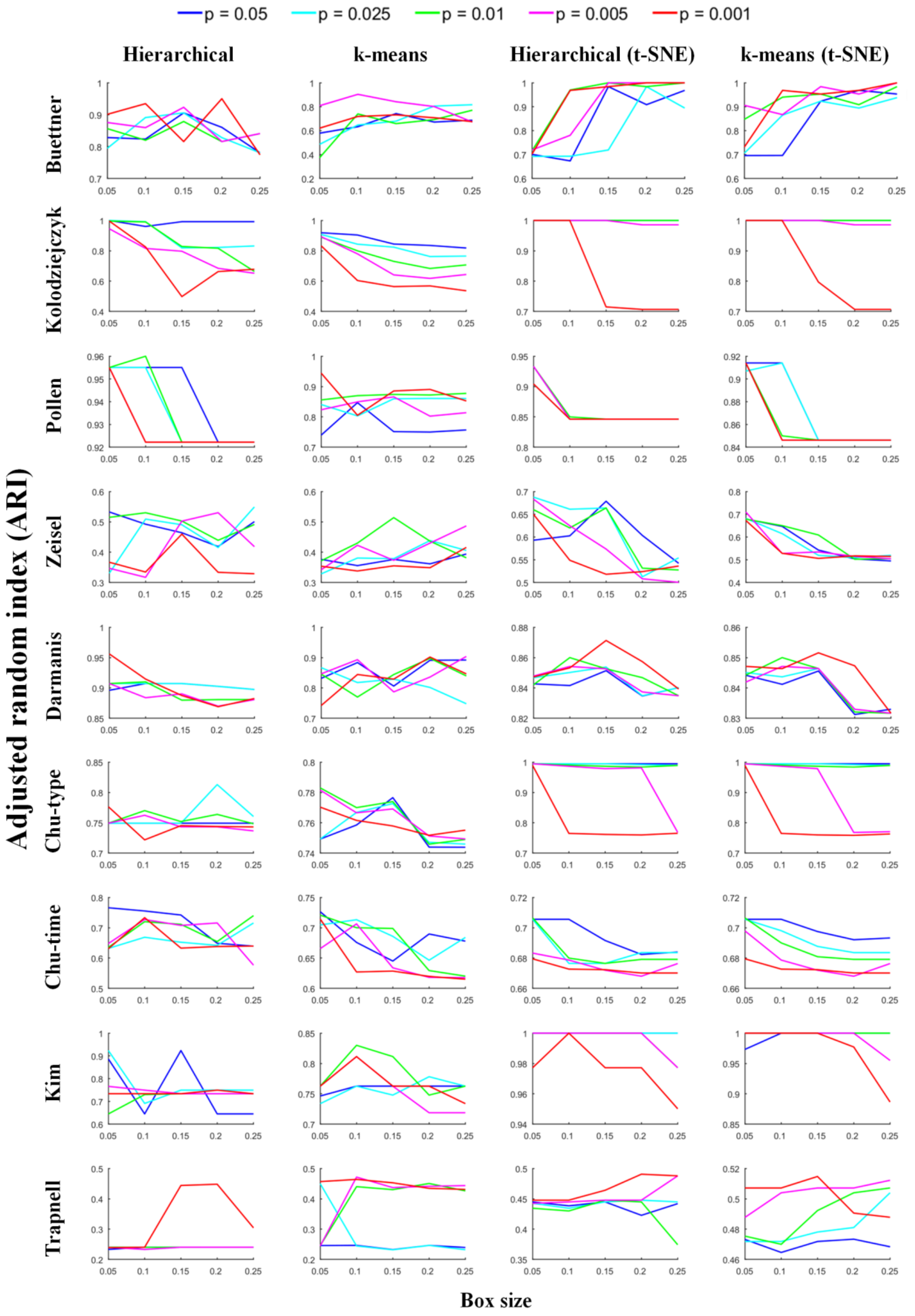

Figure S1. Comparison of different parameters of CSN. X-axis is box size and different colors represent different p-value. Y-axis is the ARI in clustering analysis. The results show that the optimum box size is about 0.1, and the optimum p-value is about 0.01, on average.

S2. The identification of 'dark genes' for T2DM with PAAD data

In clinical practice and scientific research, differentially expressed genes draw much attention in early diagnosis of disease, screening drug targets, treating diseases, and developing new drugs. However, some non-differential genes in the coding region of DNA are called "Dark Matter" [2]. Based on DMNE method, we find some 'dark genes' with-out differential expressions which are especially sensitive to DMNE score. Traditional analyses usually ignore them.

Pancreatic adenocarcinoma (PAAD) and T2DM are inter-related. The outcomes of this association are the topic of interest of a lot of prior research in this field. At present, due to the lack of corresponding prognostic data in the study of T2DM, the prognostic analysis of T2DM cannot be performed directly. Therefore, we use the data of PAAD to analyze the prognosis of T2DM, attempting to reveal the mechanism of the development of T2DM from another view.

PAAD survival data and the gene expression values in different samples are downloaded from the TCGA database. 178 samples are selected for analysis. The expression values are processed by DMNE to obtain the DMNE score. We analyze the prognosis of these 'dark genes' respectively based on gene expression and DMNE score by dividing the samples into two groups based on the median of genes expression or DMNE score. Group 1 is a group with higher value and Group 2 is a group with a lower value. Based on the result of prognosis, the 'dark genes' can be categorized into two types of molecules as a mutual biomarker for all samples. Those genes with high scores that cause poor prognosis are termed "negative dark genes", and those genes with high scores that cause good prognosis termed "positive dark genes". If "negative dark genes" appear in the DNBs, the sample's prognosis will be more negative than that of other samples. Similarly, if "positive dark genes" appear in the DNBs, the sample's prognosis will be more positive than others.

Further analysis shows that 'dark genes' are all strongly related to patients' survival based on DMNE score but not expression levels. Figure.S4 shows the survival analysis of PCOLCE, HPX, BCKDHA, IFITM1, DEPDC1 and COL1A1 results with P-values < 0.05 based on some non-differential genes in DNBs of T2DM. As shown in Figure S4, the p values of survival curves obtained from gene expression values are all greater than 0.05 (PCOLCE:0.56, HPX:0.41, BCKDHA:0.89, IFITM1:0.19, DEPDC1:0.72 COL1A1:0.93), and the p values of survival curves obtained from DMNE score are all less than 0.05 (PCOLCE:0.034, HPX:0.036, BCKDHA:0.028, IFITM1:0.031, DEPDC1:0.0041, COL1A1:0.0071). P value <0.05 is statistically significant. A higher level of DMNE score in PCOLCE and HPX is significantly related to a good prognosis, i.e., positive 'dark genes'. While a higher level of DMNE score in BCKDHA, IFITM1, DEPDC1 and COL1A1 is significantly related to poor prognosis, i.e., negative 'dark genes' (Figure 4). COL1A1 is linked to hypoglycemic activity [2]. This shows the effectiveness of the development of PAAD for the 'dark genes' in the DNBs from the side.

A large number of epidemiological studies have found that T2DM is positively associated with an increased risk of PAAD. Long-term type 2 diabetes increases the risk of PAAD by 1.5 to 2 times. Thirty to 40 percent of pancreatic cancer patients have

diabetes and 80 percent have abnormal glucose tolerance. Therefore, some 'dark genes' related with the prognosis of PAAD may play important role in the development of T2DM, such as COL1A1 is the most significant gene in the extracellular matrix receptor interaction pathway and is linked to hypoglycemic activity [3], which may affect the fluctuation of blood glucose and the prognosis of T2DM and the occurrence and development of chronic complications [4].

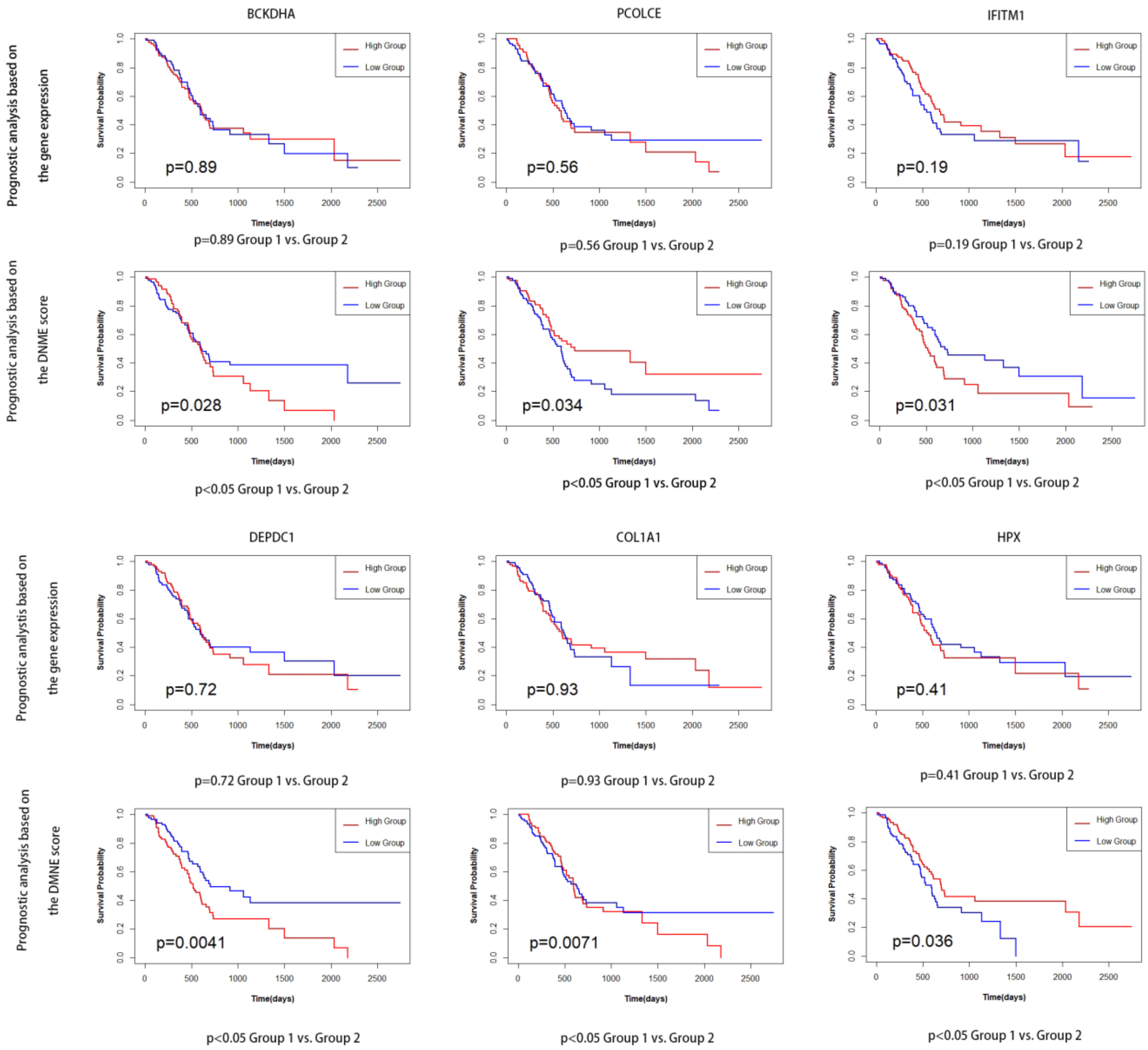

Figure S2. The comparison of prognosis analysis between gene expression and DMNE score of 'dark genes'. For T2DM, BCKDHA, PCOLCE, IFITM1, DEPDC1, COL1A1 and HPX, are 'dark genes', whose DMNE scores are more sensitive to the early-warning signal of disease deterioration, and can predict prognosis better than gene expressions. The prognosis of these 'dark genes' show that there are significant differences between survival times of the two group samples, i.e., the high value group and the low value group, based on DMNE scores rather than gene expressions.

S3. Drug target analysis for GSE13268: Rat Adipose Tissue

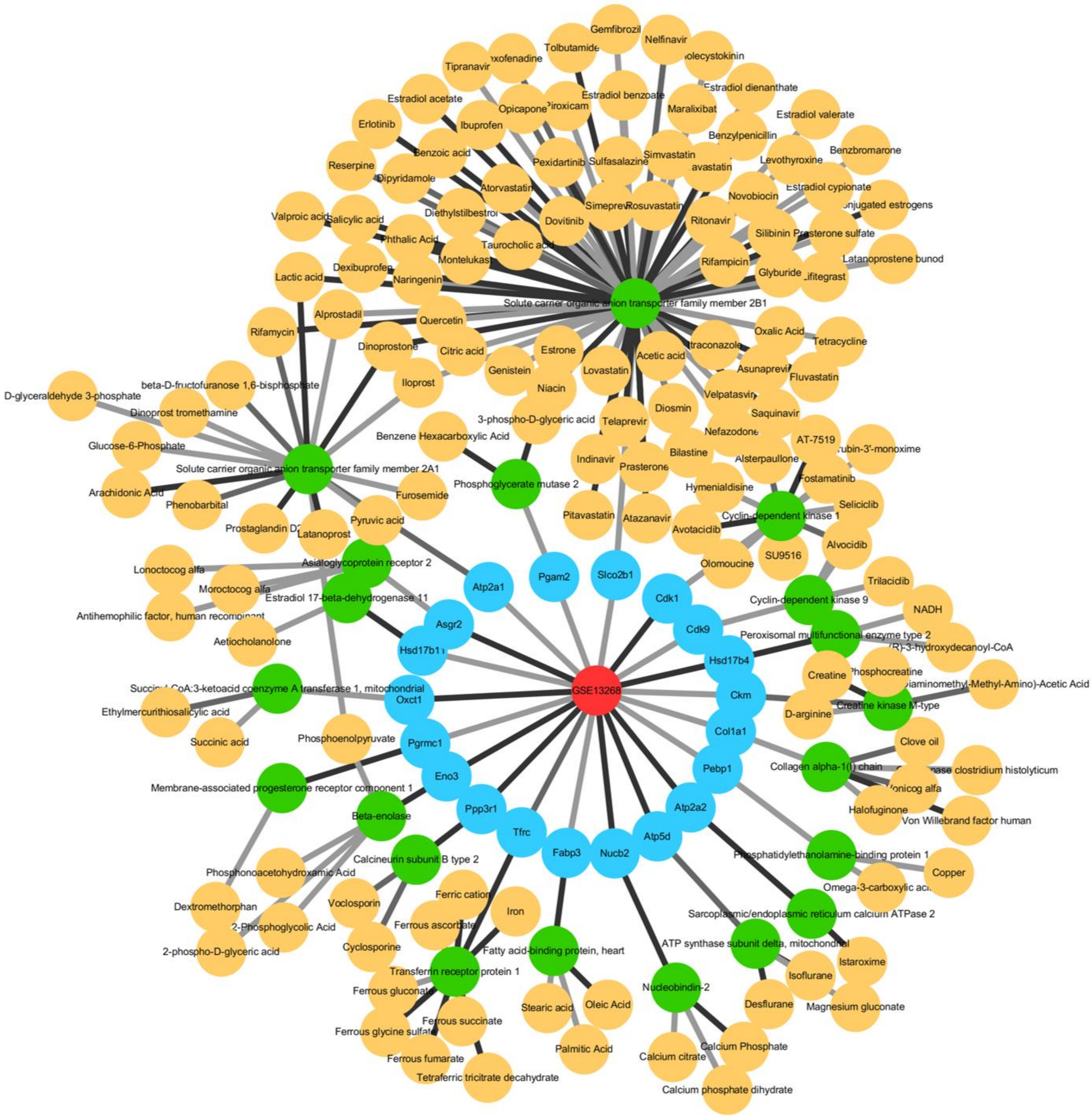

a

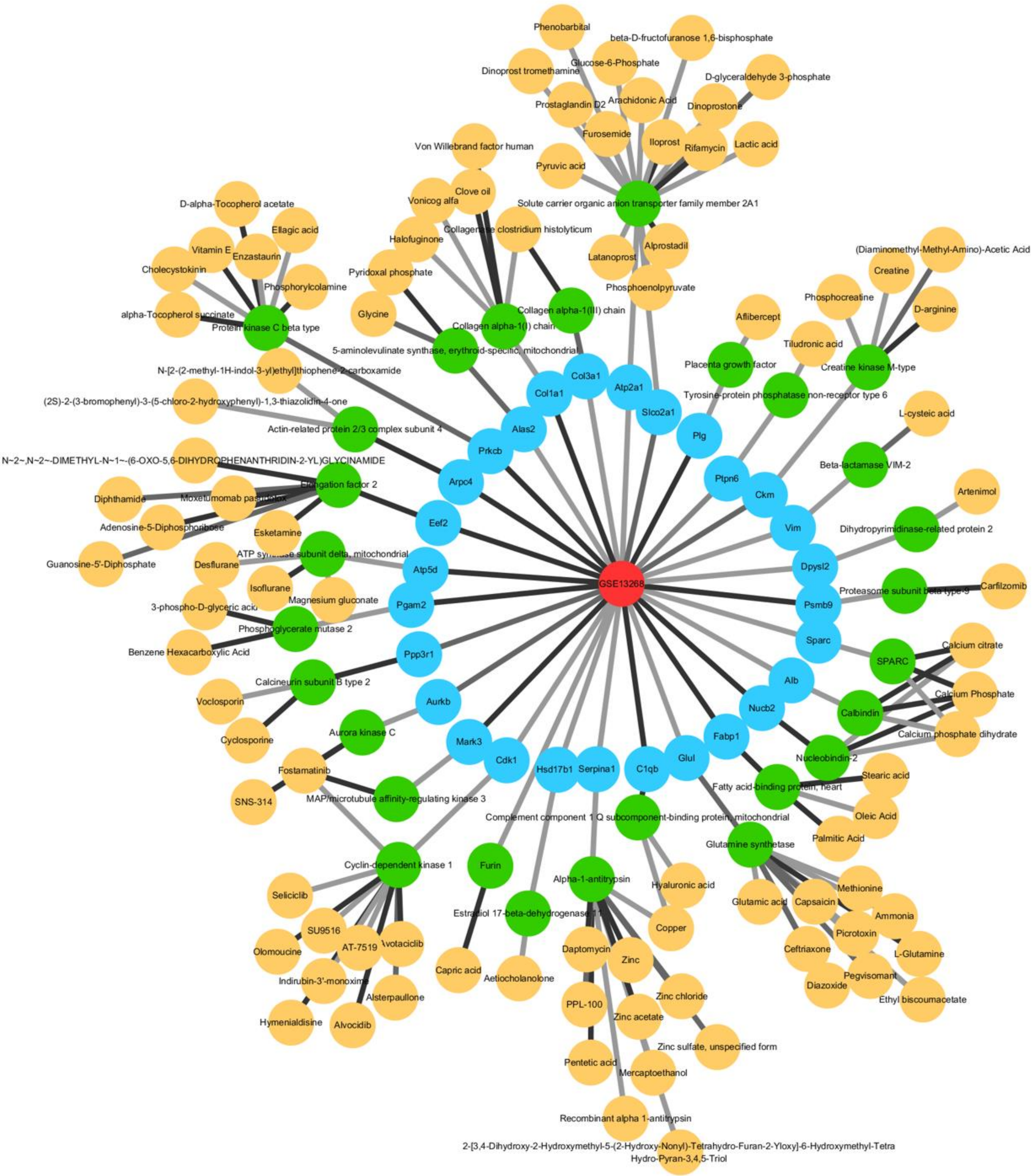
GSE13268
Col1a1
Col3a1
Atp2a1
Slco2a1
Alas2
Prkcb
Arpc4
Eef2
Atp5d
Pgam2
Ppp3r1
Aurkb
Mark3
Cdk1
Hsd17b1
Serpina1
C1qb
Glul
Fabp1
Nucb2
Alb
Sparc
Psmb9
Dpysl2
Vim
Ckm
Ptpn6
Plg
Phenobarbital
beta-D-fructofuranose 1,6-bisphosphate
Glucose-6-Phosphate
Dinoprost tromethamine
D-glyceraldehyde 3-phosphate
Prostaglandin D2
Arachidonic Acid
Dinoprostone
Furosemide
Iloprost
Rifamycin
Lactic acid
Pyruvic acid
Solute carrier organic anion transporter family member 2A1
Latanoprost
Alprostadil
Phosphoenolpyruvate
Von Willebrand factor human
Clove oil
Vonicog alfa
Collagenase clostridium histolyticum
Halofuginone
Pyridoxal phosphate
Glycine
Collagen alpha-1(III) chain
Collagen alpha-1(I) chain
5-aminolevulinate synthase, erythroid-specific, mitochondrial
D-alpha-Tocopherol acetate
Ellagic acid
Vitamin E
Enzastaurin
Cholecystokinin
Phosphorylcolamine
alpha-Tocopherol succinate
Protein kinase C beta type
N-[2-(2-methyl-1H-indol-3-yl)ethyl]thiophene-2-carboxamide
(2S)-2-(3-bromophenyl)-3-(5-chloro-2-hydroxyphenyl)-1,3-thiazolidin-4-one
Actin-related protein 2/3 complex subunit 4
N~2~,N~2~-DIMETHYL-N~1~-(6-OXO-5,6-DIHYDROPHENANTHRIDIN-2-YL)GLYCINAMIDE
Elongation factor 2
Diphthamide
Adenosine-5-Diphosphoribose
Esketamine
Guanosine-5'-Diphosphate
ATP synthase subunit delta, mitochondrial
Desflurane
Isoflurane
Magnesium gluconate
3-phospho-D-glyceric acid
Phosphoglycerate mutase 2
Benzene Hexacarboxylic Acid
Calcineurin subunit B type 2
Voclosporin
Cyclosporine
Aurora kinase C
Fostamatinib
SNS-314
MAP/microtubule affinity-regulating kinase 3
Cyclin-dependent kinase 1
Seliciclib
SU9516
Olomoucine
AT-7519
Alvocidib
Indirubin-3'-monoxime
Alsterpaullone
Hymenialdisine
Alvocidib
Furin
Capric acid
Estradiol 17-beta-dehydrogenase 1
Aetiocholanolone
Alpha-1-antitrypsin
Daptomycin
Zinc
PPL-100
Zinc chloride
Zinc acetate
Zinc sulfate, unspecified form
Pentetic acid
Mercaptoethanol
Recombinant alpha 1-antitrypsin
2-[3,4-Dihydroxy-2-Hydroxymethyl-5-(2-Hydroxy-Nonyl)-Tetrahydro-Furan-2-Yloxy]-6-Hydroxymethyl-Tetra
Hydro-Pyran-3,4,5-Triol
Complement component 1 Q subcomponent-binding protein, mitochondrial
Hyaluronic acid
Copper
Glutamine synthetase
Glutamic acid
Capsaicin
Methionine
Ammonia
Picrotoxin
Ceftriaxone
L-Glutamine
Pegvisomant
Diazoxide
Ethyl biscoumacetate
Fatty acid-binding protein, heart
Stearic acid
Oleic Acid
Palmitic Acid
Nucleobindin-2
Calbindin
SPARC
Calcium citrate
Calcium Phosphate
Calcium phosphate dihydrate
Proteasome subunit beta type-9
Carfilzomib
Dihydropyrimidinase-related protein 2
Artenimol
Beta-lactamase VIM-2
L-cysteic acid
Tyrosine-protein phosphatase non-receptor type 6
Tiludronic acid
Placenta growth factor
Aflibercept
Creatine kinase M-type
Phosphocreatine
Creatine
(Diaminomethyl-Methyl-Amino)-Acetic Acid
D-arginine

b

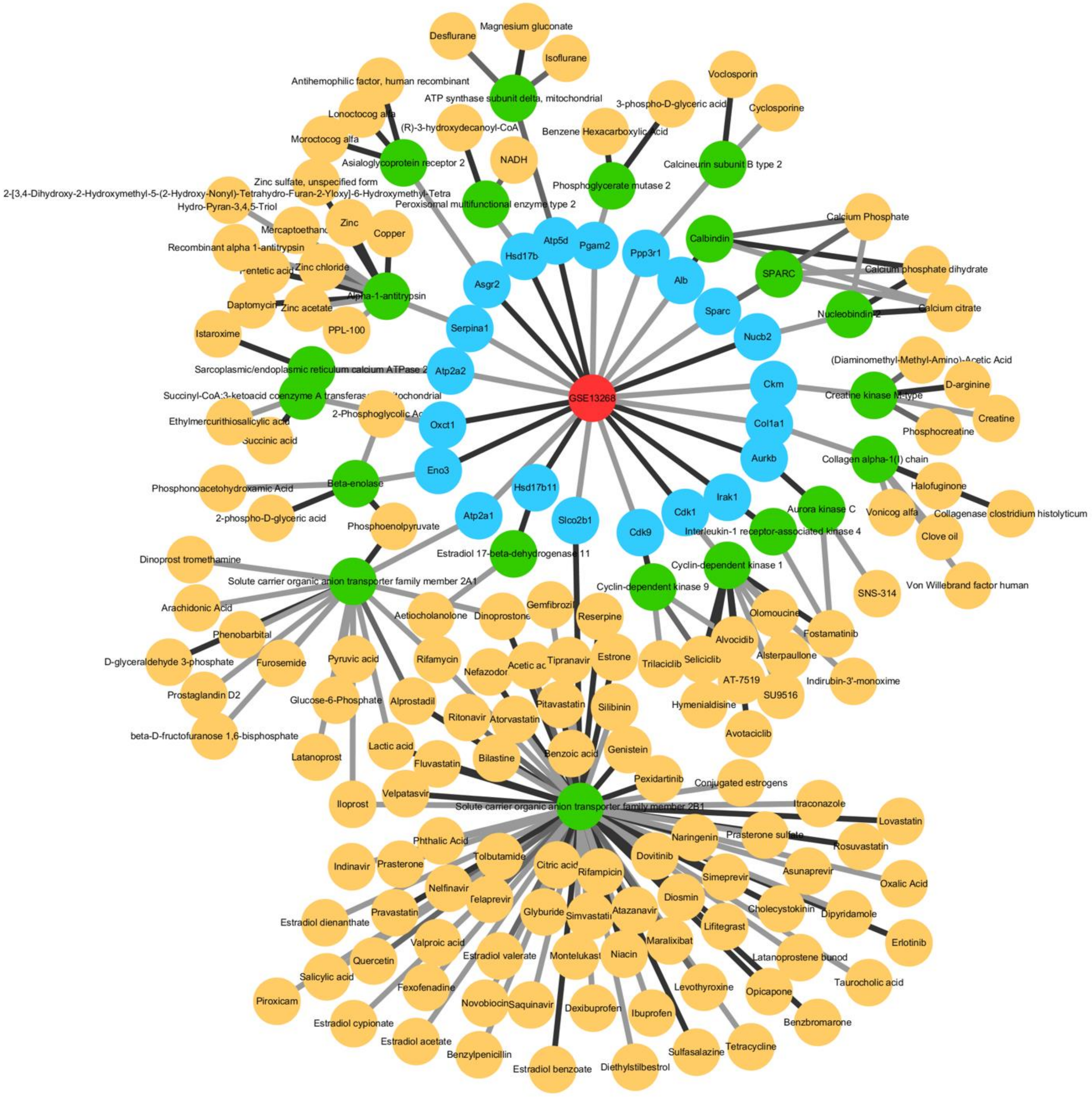
Magnesium gluconate
Desflurane
Isoflurane
Antihemophilic factor, human recombinant
ATP synthase subunit delta, mitochondrial
Voclosporin
Lonoctocog alfa
(R)-3-hydroxydecanoyl-CoA
3-phospho-D-glyceric acid
Cyclosporine
Moroctocog alfa
Benzene Hexacarboxylic Acid
NADH
Asialoglycoprotein receptor 2
Calcineurin subunit B type 2
Zinc sulfate, unspecified form
Phosphoglycerate mutase 2
2-[3,4-Dihydroxy-2-Hydroxymethyl-5-(2-Hydroxy-Nonyl)-Tetrahydro-Furan-2-Yloxy]-6-Hydroxymethyl-Tetra
Peroxisomal multifunctional enzyme type 2
Hydro-Pyran-3,4,5-Triol
Zinc
Calcium Phosphate
Mercaptoethanol
Copper
Atp5d
Calbindin
Recombinant alpha 1-antitrypsin
Hsd17b
Pgam2
Ppp3r1
Zinc chloride
SPARC
Calcium phosphate dihydrate
Asgr2
Alb
Alpha-1-antitrypsin
Daptomycin
Zinc acetate
Sparc
Nucleobindin-2
Calcium citrate
Serpina1
Istaroxime
PPL-100
Nucb2
(Diaminomethyl-Methyl-Amino)-Acetic Acid
Sarcoplasmic/endoplasmic reticulum calcium ATPase 2
Atp2a2
Ckm
D-arginine
GSE13268
Creatine kinase M-type
Succinyl-CoA:3-ketoacid coenzyme A transferase
Creatine
Ethylmercurithiosalicylic acid
2-Phosphoglycolic Acid
Oxct1
Col1a1
Phosphocreatine
Succinic acid
Aurkb
Collagen alpha-1(I) chain
Eno3
Phosphonoacetohydroxamic Acid
Beta-enolase
Halofuginone
Hsd17b11
Irak1
Aurora kinase C
2-phospho-D-glyceric acid
Cdk1
Vonicog alfa
Collagenase clostridium histolyticum
Atp2a1
Slco2b1
Phosphoenolpyruvate
Interleukin-1 receptor-associated kinase 4
Cdk9
Clove oil
Estradiol 17-beta-dehydrogenase 11
Dinoprost tromethamine
Cyclin-dependent kinase 1
Solute carrier organic anion transporter family member 2A1
Cyclin-dependent kinase 9
SNS-314
Von Willebrand factor human
Gemfibrozil
Arachidonic Acid
Aetiocholanolone
Dinoprostone
Reserpine
Olomoucine
Phenobarbital
Fostamatinib
Alvocidib
D-glyceraldehyde 3-phosphate
Furosemide
Pyruvic acid
Rifamycin
Estrone
Tipranavir
Trilaciclib
Seliciclib
Alsterpaullone
Nefazodone
Indirubin-3'-monoxime
AT-7519
Prostaglandin D2
Glucose-6-Phosphate
Alprostadil
Pitavastatin
Silibinin
SU9516
Hymenialdisine
Ritonavir
Atorvastatin
beta-D-fructofuranose 1,6-bisphosphate
Avotaciclib
Lactic acid
Genistein
Latanoprost
Bilastine
Benzoic acid
Fluvastatin
Pexidartinib
Conjugated estrogens
Iloprost
Velpatasvir
Itraconazole
Solute carrier organic anion transporter family member 2B1
Lovastatin
Naringenin
Prasterone sulfate
Phthalic Acid
Rosuvastatin
Indinavir
Tolbutamide
Prasterone
Dovitinib
Citric acid
Asunaprevir
Rifampicin
Oxalic Acid
Simeprevir
Nelfinavir
Telaprevir
Diosmin
Cholecystokinin
Dipyridamole
Pravastatin
Glyburide
Atazanavir
Simvastatin
Estradiol dienanthate
Lifitegrast
Maralixibat
Erlotinib
Valproic acid
Quercetin
Estradiol valerate
Montelukast
Niacin
Latanoprostene bunod
Salicylic acid
Taurocholic acid
Fexofenadine
Levothyroxine
Opicapone
Piroxicam
Novobiocin
Saquinavir
Dexibuprofen
Ibuprofen
Estradiol cypionate
Benzbromarone
Estradiol acetate
Tetracycline
Sulfasalazine
Benzylpenicillin
Estradiol benzoate
Diethylstilbestrol

c

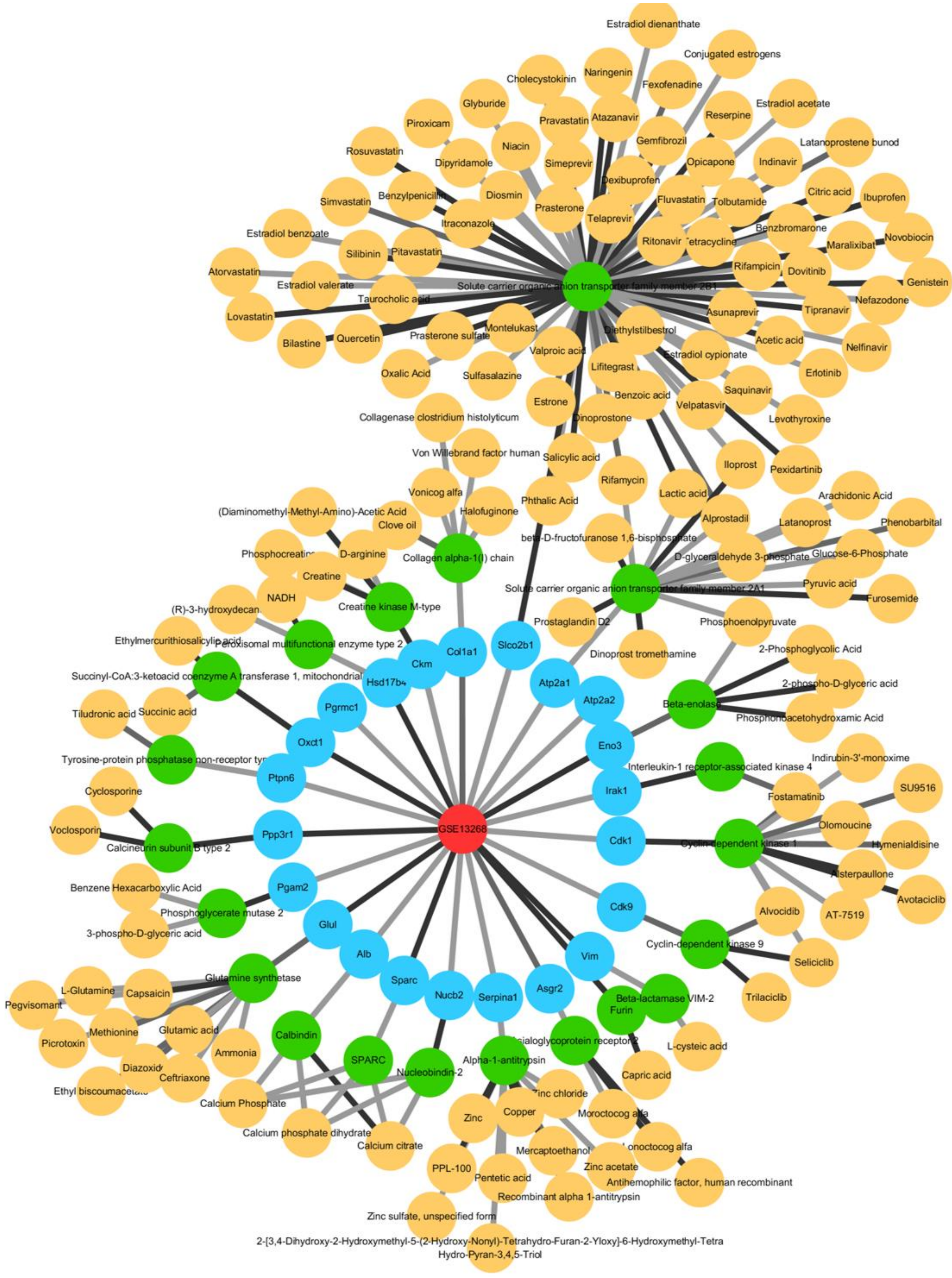
GSE13268
Estradiol dienanthate
Conjugated estrogens
Cholecystokinin
Naringenin
Fexofenadine
Glyburide
Estradiol acetate
Piroxicam
Pravastatin
Atazanavir
Reserpine
Niacin
Gemfibrozil
Latanoprostene bunod
Rosuvastatin
Dipyridamole
Simeprevir
Opicapone
Indinavir
Dexibuprofen
Diosmin
Citric acid
Ibuprofen
Simvastatin
Fluvastatin
Tolbutamide
Prasterone
Telaprevir
Itraconazole
Benzbromarone
Novobiocin
Estradiol benzoate
Ritonavir
Maralixibat
Silibinin
Pitavastatin
Rifampicin
Dovitinib
Genistein
Atorvastatin
Estradiol valerate
Taurocholic acid
Nefazodone
Lovastatin
Asunaprevir
Tipranavir
Montelukast
Prasterone sulfate
Bilastine
Quercetin
Acetic acid
Nelfinavir
Valproic acid
Estradiol cypionate
Oxalic Acid
Sulfasalazine
Erlotinib
Benzoic acid
Saquinavir
Estrone
Velpatasvir
Dinoprostone
Levothyroxine
Collagenase clostridium histolyticum
Von Willebrand factor human
Salicylic acid
Iloprost
Pexidartinib
Rifamycin
Vonicog alfa
Phthalic Acid
Lactic acid
Arachidonic Acid
(Diaminomethyl-Methyl-Amino)-Acetic Acid
Halofuginone
Alprostadil
Clove oil
Latanoprost
Phenobarbital
D-arginine
Collagen alpha-1(I) chain
Glucose-6-Phosphate
Creatine
Pyruvic acid
NADH
Furosemide
Creatine kinase M-type
Prostaglandin D2
Phosphoenolpyruvate
Peroxisomal multifunctional enzyme type 2
Col1a1
Slco2b1
Dinoprost tromethamine
2-Phosphoglycolic Acid
Ckm
Hsd17b4
Atp2a1
2-phospho-D-glyceric acid
Atp2a2
Beta-enolase
Tiludronic acid
Succinic acid
Pgrmc1
Phosphonoacetohydroxamic Acid
Oxct1
Eno3
Indirubin-3'-monoxime
Ptpn6
Fostamatinib
Cyclosporine
Irak1
SU9516
Olomoucine
Voclosporin
Ppp3r1
Cdk1
Hymenialdisine
Calcineurin subunit B type 2
Alsterpaullone
Benzene Hexacarboxylic Acid
Pgam2
Avotaciclib
Phosphoglycerate mutase 2
Alvocidib
AT-7519
Cdk9
3-phospho-D-glyceric acid
Glul
Cyclin-dependent kinase 9
Alb
Vim
Seliciclib
Glutamine synthetase
Sparc
L-Glutamine
Capsaicin
Nucb2
Serpina1
Asgr2
Beta-lactamase VIM-2
Trilaciclib
Pegvisomant
Furin
Methionine
Glutamic acid
Calbindin
L-cysteic acid
Picrotoxin
Ammonia
SPARC
Alpha-1-antitrypsin
Diazoxide
Nucleobindin-2
Capric acid
Ceftriaxone
Calcium Phosphate
Zinc
Copper
Calcium phosphate dihydrate
Calcium citrate
Mercaptoethanol
Zinc acetate
PPL-100
Pentetic acid
Antihemophilic factor, human recombinant
Recombinant alpha 1-antitrypsin
Zinc sulfate, unspecified form
2-[3,4-Dihydroxy-2-Hydroxymethyl-5-(2-Hydroxy-Nonyl)-Tetrahydro-Furan-2-Yloxy]-6-Hydroxymethyl-Tetra
Hydro-Pyran-3,4,5-Triol

d

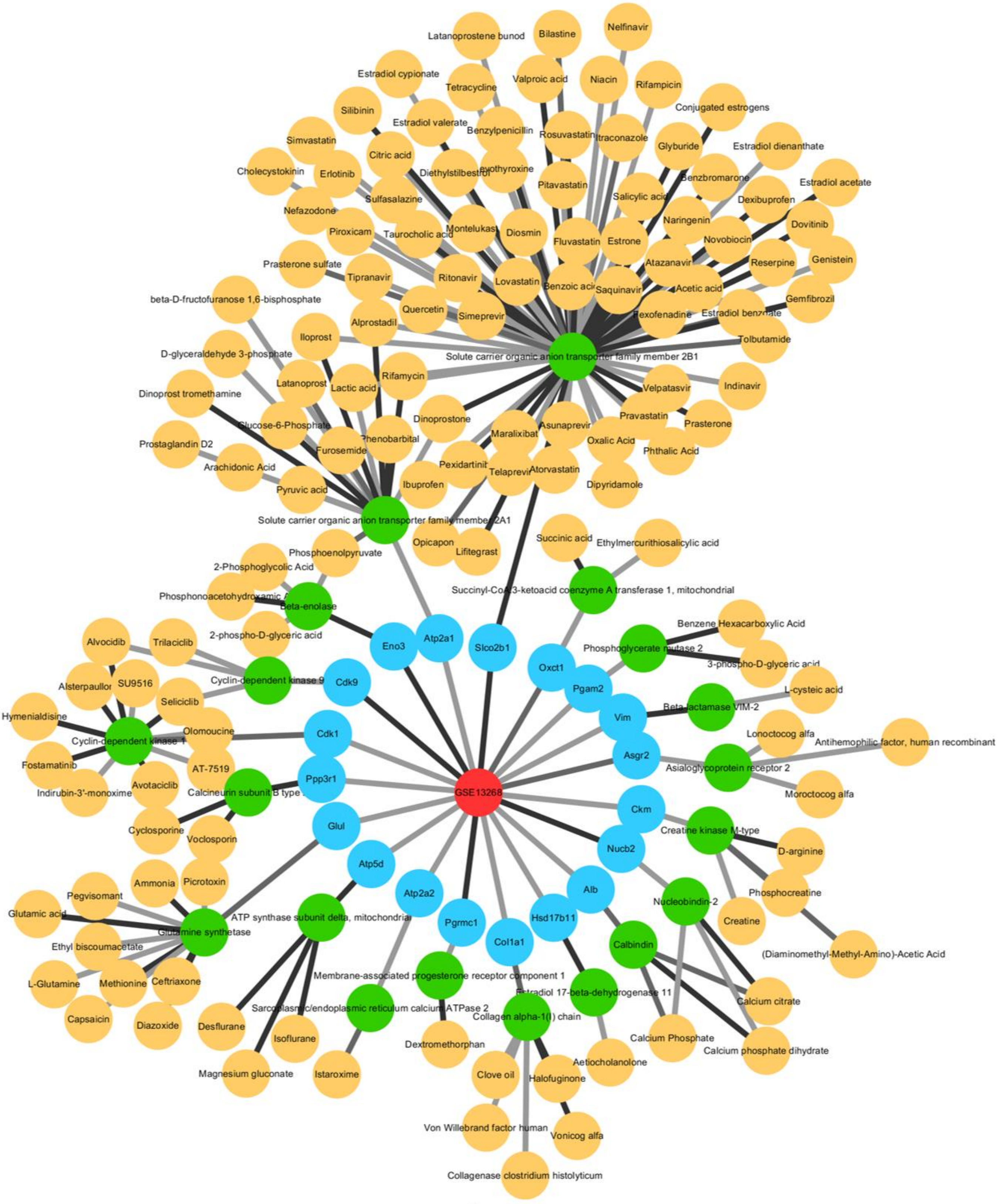

e

Figure S3: The drug target analysis for GSE13268, involving rat adipose tissue. (a) Drug targets, the corresponding proteins and targeted drugs at (a) stage I; (b) stage II; (c)

stage III; (d) stage IV; (e) stage V.

In the drug target of the second phase of GSE13268, CKM, or creatine kinase, is a target protein sought from mouse skeletal muscle and is associated with T2D-associated energy metabolism. CKM binds to 16:0/16:0-PA, with the highest affinity among PA binding proteins reported to date [5]. Col1a1 is the most important gene in extracellular matrix receptor interactions and has been identified to be associated with hypoglycemic activity. Therefore, in terms of alleviating T2D, Col1a1 can serve as a new potential therapeutic target [3].

S4. Drug target analysis for GSE13269: Rat Gastrocnemius Muscle Tissue

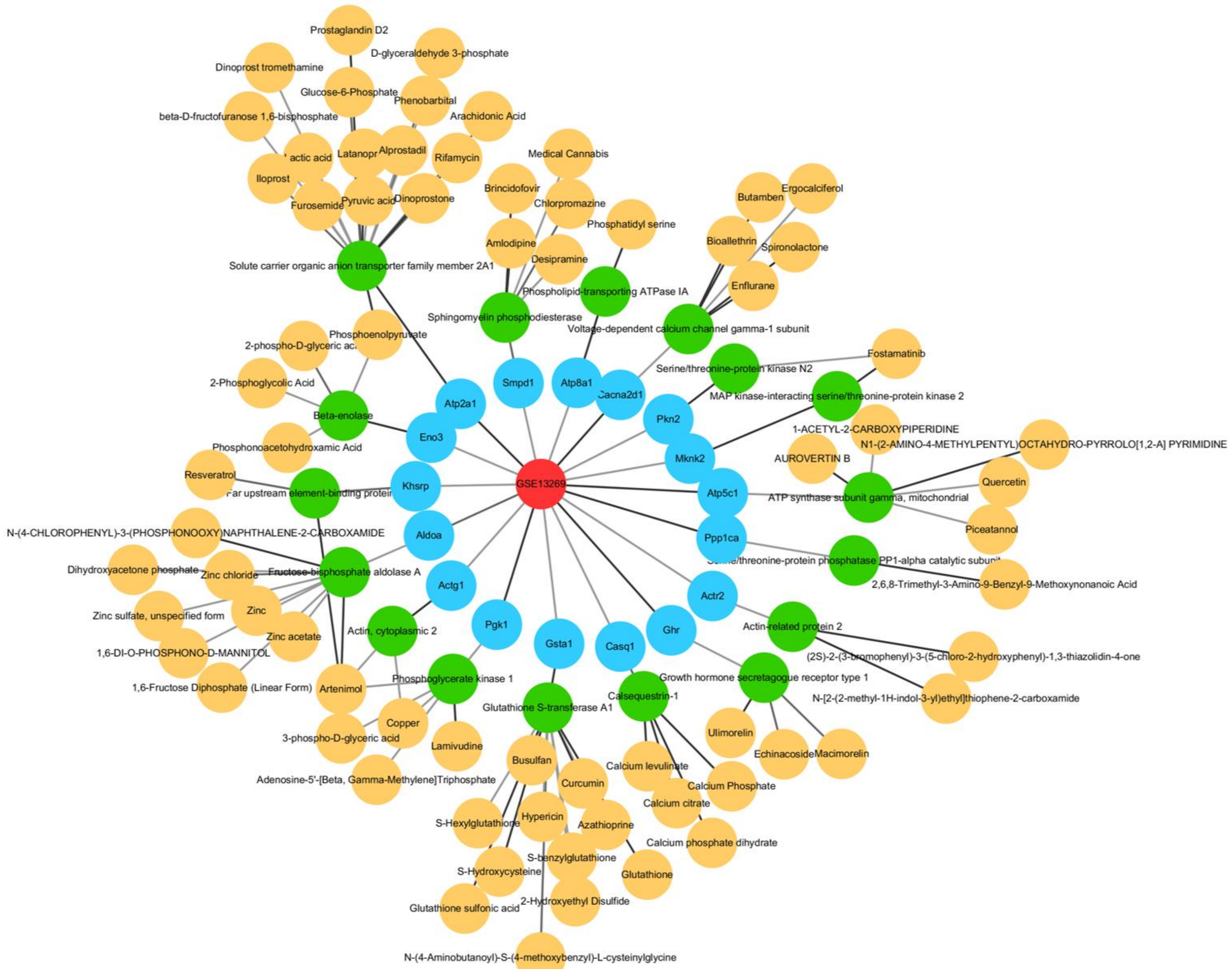

a

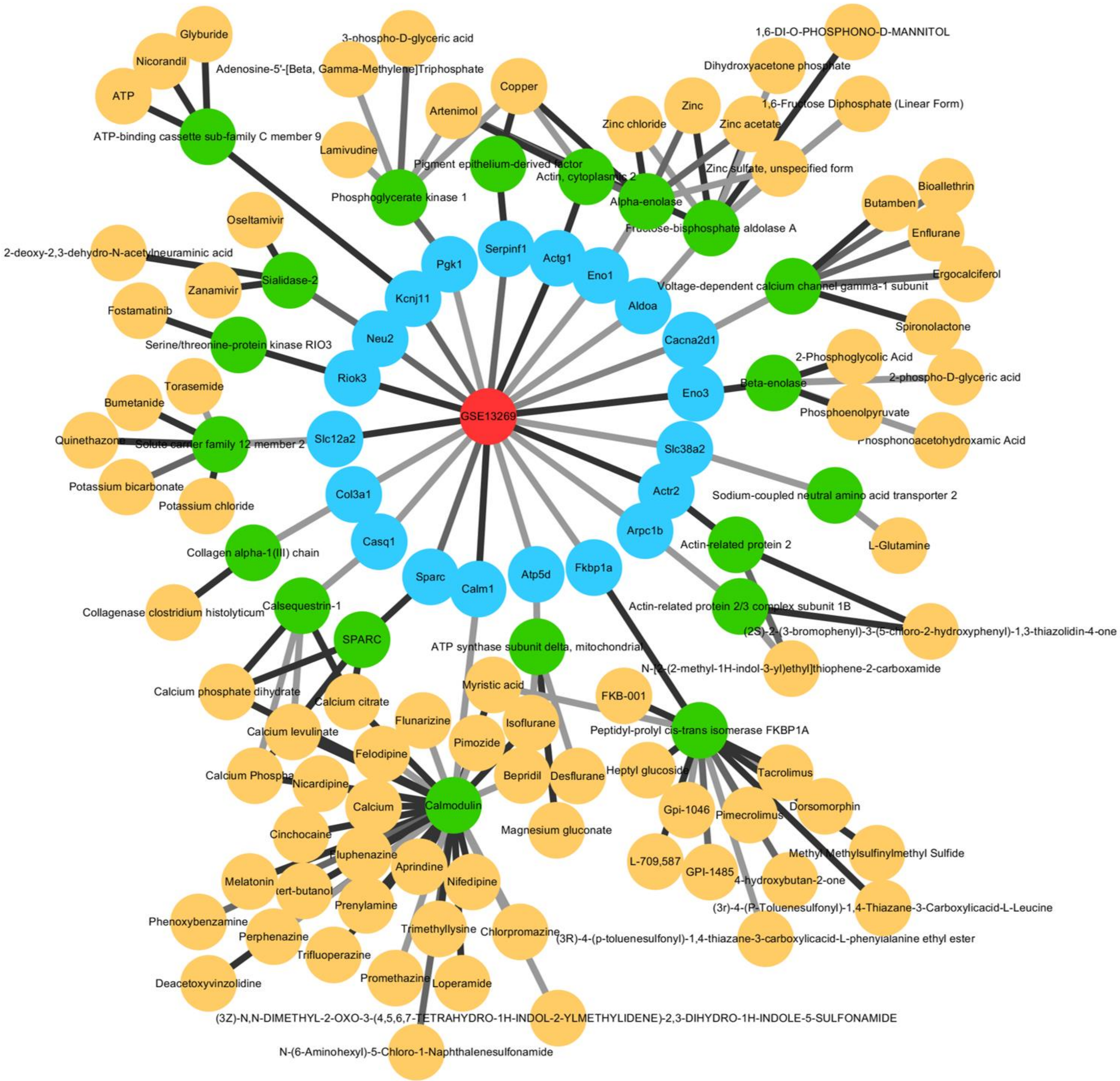
GSE13269
Pgk1
Serpinf1
Actg1
Eno1
Aldoa
Kcnj11
Neu2
Riok3
Slc12a2
Col3a1
Casq1
Sparc
Calm1
Atp5d
Fkbp1a
Arpc1b
Actr2
Slc38a2
Eno3
Cacna2d1
ATP-binding cassette sub-family C member 9
Glyburide
Nicorandil
ATP
Adenosine-5'-[Beta, Gamma-Methylene]Triphosphate
3-phospho-D-glyceric acid
Lamivudine
Artenimol
Copper
Phosphoglycerate kinase 1
Pigment epithelium-derived factor
Actin, cytoplasmic 2
Alpha-enolase
Fructose-bisphosphate aldolase A
Zinc chloride
Zinc
Zinc acetate
Zinc sulfate, unspecified form
Dihydroxyacetone phosphate
1,6-DI-O-PHOSPHONO-D-MANNITOL
1,6-Fructose Diphosphate (Linear Form)
Oseltamivir
2-deoxy-2,3-dehydro-N-acetylneuraminic acid
Zanamivir
Sialidase-2
Fostamatinib
Serine/threonine-protein kinase RIO3
Butamben
Bioallethrin
Enflurane
Ergocalciferol
Voltage-dependent calcium channel gamma-1 subunit
Spironolactone
2-Phosphoglycolic Acid
Beta-enolase
2-phospho-D-glyceric acid
Phosphoenolpyruvate
Phosphonoacetohydroxamic Acid
Torasemide
Bumetanide
Quinethazone
Solute carrier family 12 member 2
Potassium bicarbonate
Potassium chloride
Sodium-coupled neutral amino acid transporter 2
L-Glutamine
Collagen alpha-1(III) chain
Collagenase clostridium histolyticum
Calsequestrin-1
SPARC
Actin-related protein 2
Actin-related protein 2/3 complex subunit 1B
(2S)-2-(3-bromophenyl)-3-(5-chloro-2-hydroxyphenyl)-1,3-thiazolidin-4-one
ATP synthase subunit delta, mitochondrial
N-[2-(2-methyl-1H-indol-3-yl)ethyl]thiophene-2-carboxamide
Calcium phosphate dihydrate
Calcium citrate
Myristic acid
FKB-001
Calcium levulinate
Flunarizine
Isoflurane
Peptidyl-prolyl cis-trans isomerase FKBP1A
Felodipine
Pimozide
Nicardipine
Bepridil
Desflurane
Heptyl glucoside
Tacrolimus
Calcium
Calmodulin
Gpi-1046
Pimecrolimus
Dorsomorphin
Magnesium gluconate
Cinchocaine
Fluphenazine
Aprindine
Nifedipine
L-709,587
GPI-1485
4-hydroxybutan-2-one
Methyl Methylsulfinylmethyl Sulfide
Melatonin
tert-butanol
Prenylamine
(3r)-4-(P-Toluenesulfonyl)-1,4-Thiazane-3-Carboxylicacid-L-Leucine
Phenoxybenzamine
Trimethyllysine
Chlorpromazine
(3R)-4-(p-toluenesulfonyl)-1,4-thiazane-3-carboxylicacid-L-phenylalanine ethyl ester
Perphenazine
Trifluoperazine
Promethazine
Loperamide
Deacetoxyvinzolidine
(3Z)-N,N-DIMETHYL-2-OXO-3-(4,5,6,7-TETRAHYDRO-1H-INDOL-2-YLMETHYLIDENE)-2,3-DIHYDRO-1H-INDOLE-5-SULFONAMIDE
N-(6-Aminohexyl)-5-Chloro-1-Naphthalenesulfonamide

b

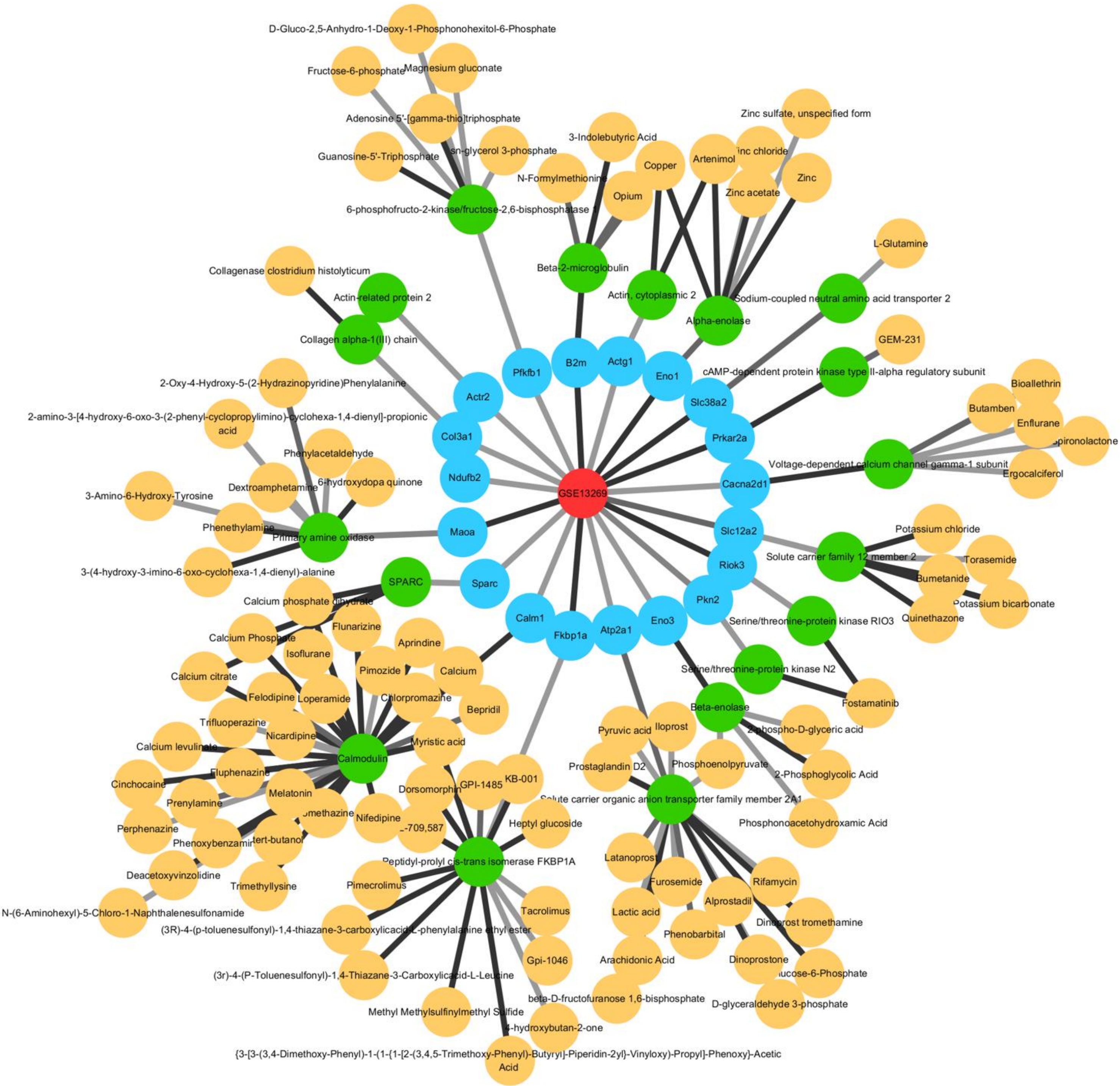
GSE13269
D-Gluco-2,5-Anhydro-1-Deoxy-1-Phosphonohexitol-6-Phosphate
Fructose-6-phosphate
Magnesium gluconate
Adenosine 5'-[gamma-thio]triphosphate
Guanosine-5'-Triphosphate
sn-glycerol 3-phosphate
6-phosphofructo-2-kinase/fructose-2,6-bisphosphatase 1
3-Indolebutyric Acid
N-Formylmethionine
Copper
Opium
Artenimol
Zinc sulfate, unspecified form
Zinc acetate
Zinc
Beta-2-microglobulin
Actin, cytoplasmic 2
Alpha-enolase
Sodium-coupled neutral amino acid transporter 2
L-Glutamine
GEM-231
cAMP-dependent protein kinase type II-alpha regulatory subunit
Bioallethrin
Butamben
Enflurane
Voltage-dependent calcium channel gamma-1 subunit
Ergocalciferol
Collagenase clostridium histolyticum
Actin-related protein 2
Collagen alpha-1(III) chain
2-Oxy-4-Hydroxy-5-(2-Hydrazinopyridine)Phenylalanine
2-amino-3-[4-hydroxy-6-oxo-3-(2-phenyl-cyclopropylimino)-cyclohexa-1,4-dienyl]-propionic acid
Phenylacetaldehyde
Dextroamphetamine
6-hydroxydopa quinone
3-Amino-6-Hydroxy-Tyrosine
Phenethylamine
Primary amine oxidase
3-(4-hydroxy-3-imino-6-oxo-cyclohexa-1,4-dienyl)-alanine
Pfkfb1
B2m
Actg1
Eno1
Actr2
Slc38a2
Col3a1
Prkar2a
Ndufb2
Cacna2d1
Maoa
Slc12a2
Riok3
Sparc
Pkn2
Calm1
Fkbp1a
Atp2a1
Eno3
SPARC
Potassium chloride
Solute carrier family 12 member 2
Torasemide
Bumetanide
Potassium bicarbonate
Quinethazone
Serine/threonine-protein kinase RIO3
Serine/threonine-protein kinase N2
Fostamatinib
Beta-enolase
2-phospho-D-glyceric acid
2-Phosphoglycolic Acid
Phosphonoacetohydroxamic Acid
Calcium phosphate dihydrate
Flunarizine
Calcium Phosphate
Isoflurane
Aprindine
Pimozide
Calcium
Calcium citrate
Felodipine
Loperamide
Chlorpromazine
Bepridil
Trifluoperazine
Nicardipine
Myristic acid
Calcium levulinate
Calmodulin
Fluphenazine
Cinchocaine
Melatonin
Prenylamine
Perphenazine
Nifedipine
Phenoxybenzamine
Deacetoxyvinzolidine
Trimethyllysine
N-(6-Aminohexyl)-5-Chloro-1-Naphthalenesulfonamide
Dorsomorphin
GPI-1485
KB-001
Heptyl glucoside
Peptidyl-prolyl cis-trans isomerase FKBP1A
Pimecrolimus
Tacrolimus
(3R)-4-(p-toluenesulfonyl)-1,4-thiazane-3-carboxylicacid-L-phenylalanine ethyl ester
Gpi-1046
(3r)-4-(P-Toluenesulfonyl)-1,4-Thiazane-3-Carboxylicacid-L-Leucine
Methyl Methylsulfinylmethyl Sulfide
4-hydroxybutan-2-one
{3-[3-(3,4-Dimethoxy-Phenyl)-1-(1-{1-[2-(3,4,5-Trimethoxy-Phenyl)-Butyryl]-Piperidin-2yl}-Vinyloxy)-Propyl]-Phenoxy}-Acetic Acid
Pyruvic acid
Iloprost
Prostaglandin D2
Phosphoenolpyruvate
Solute carrier organic anion transporter family member 2A1
Latanoprost
Furosemide
Rifamycin
Alprostadil
Lactic acid
Dinoprost tromethamine
Phenobarbital
Arachidonic Acid
Dinoprostone
beta-D-fructofuranose 1,6-bisphosphate
D-glyceraldehyde 3-phosphate

c

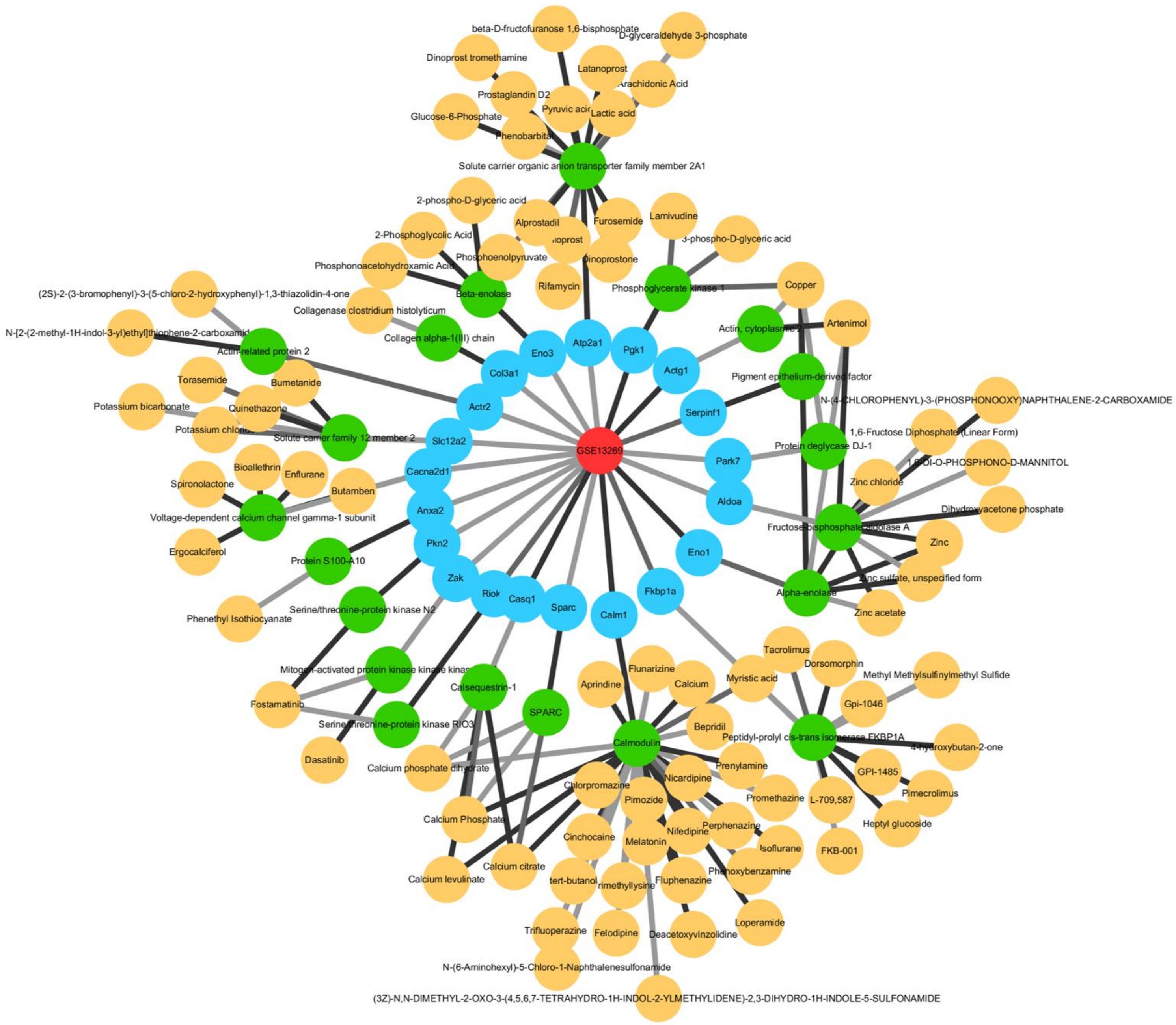

beta-D-fructofuranose 1,6-bisphosphate
D-glyceraldehyde 3-phosphate
Dinoprost tromethamine
Latanoprost
Arachidonic Acid
Prostaglandin D2
Pyruvic acid
Lactic acid
Glucose-6-Phosphate
Phenobarbital
Solute carrier organic anion transporter family member 2A1
2-phospho-D-glyceric acid
Alprostadil
Furosemide
Lamivudine
2-Phosphoglycolic Acid
Phosphoenolpyruvate
Dinoprostone
3-phospho-D-glyceric acid
Phosphonoacetohydroxamic Acid
Rifamycin
Phosphoglycerate kinase 1
Copper
Beta-enolase
(2S)-2-(3-bromophenyl)-3-(5-chloro-2-hydroxyphenyl)-1,3-thiazolidin-4-one
Collagenase clostridium histolyticum
Collagen alpha-1(III) chain
Artenimol
N-[2-(2-methyl-1H-indol-3-yl)ethyl]thiophene-2-carboxamide
Actin-related protein 2
Atp2a1
Pgk1
Eno3
Actg1
Col3a1
Torasemide
Bumetanide
Pigment epithelium-derived factor
Actr2
N-(4-CHLOROPHENYL)-3-(PHOSPHONOOXY)NAPHTHALENE-2-CARBOXAMIDE
Potassium bicarbonate
Quinethazone
Serpinf1
1,6-Fructose Diphosphate (Linear Form)
Solute carrier family 12 member 2
Slc12a2
GSE13269
Protein deglycase DJ-1
Park7
1,6-DI-O-PHOSPHONO-D-MANNITOL
Bioallethrin
Enflurane
Cacna2d1
Spironolactone
Zinc chloride
Butamben
Aldoa
Anxa2
Dihydroxyacetone phosphate
Voltage-dependent calcium channel gamma-1 subunit
Fructose-bisphosphate aldolase A
Pkn2
Eno1
Zinc
Ergocalciferol
Protein S100-A10
Zak
Fkbp1a
Zinc sulfate, unspecified form
Casq1
Sparc
Calm1
Alpha-enolase
Zinc acetate
Serine/threonine-protein kinase N2
Phenethyl Isothiocyanate
Tacrolimus
Dorsomorphin
Flunarizine
Methyl Methylsulfinylmethyl Sulfide
Aprindine
Calcium
Myristic acid
Gpi-1046
Calsequestrin-1
Fostamatinib
SPARC
Bepridil
Serine/threonine-protein kinase RIO3
Calmodulin
4-hydroxybutan-2-one
Dasatinib
Prenylamine
GPI-1485
Calcium phosphate dihydrate
Nicardipine
Chlorpromazine
Pimecrolimus
Promethazine
L-709,587
Pimozide
Calcium Phosphate
Heptyl glucoside
Perphenazine
Nifedipine
Cinchocaine
Isoflurane
Melatonin
FKB-001
Calcium citrate
Phenoxybenzamine
Calcium levulinate
tert-butanol
Fluphenazine
Loperamide
Trifluoperazine
Felodipine
Deacetoxyvinzolidine
N-(6-Aminohexyl)-5-Chloro-1-Naphthalenesulfonamide
(3Z)-N,N-DIMETHYL-2-OXO-3-(4,5,6,7-TETRAHYDRO-1H-INDOL-2-YLMETHYLIDENE)-2,3-DIHYDRO-1H-INDOLE-5-SULFONAMIDE

d

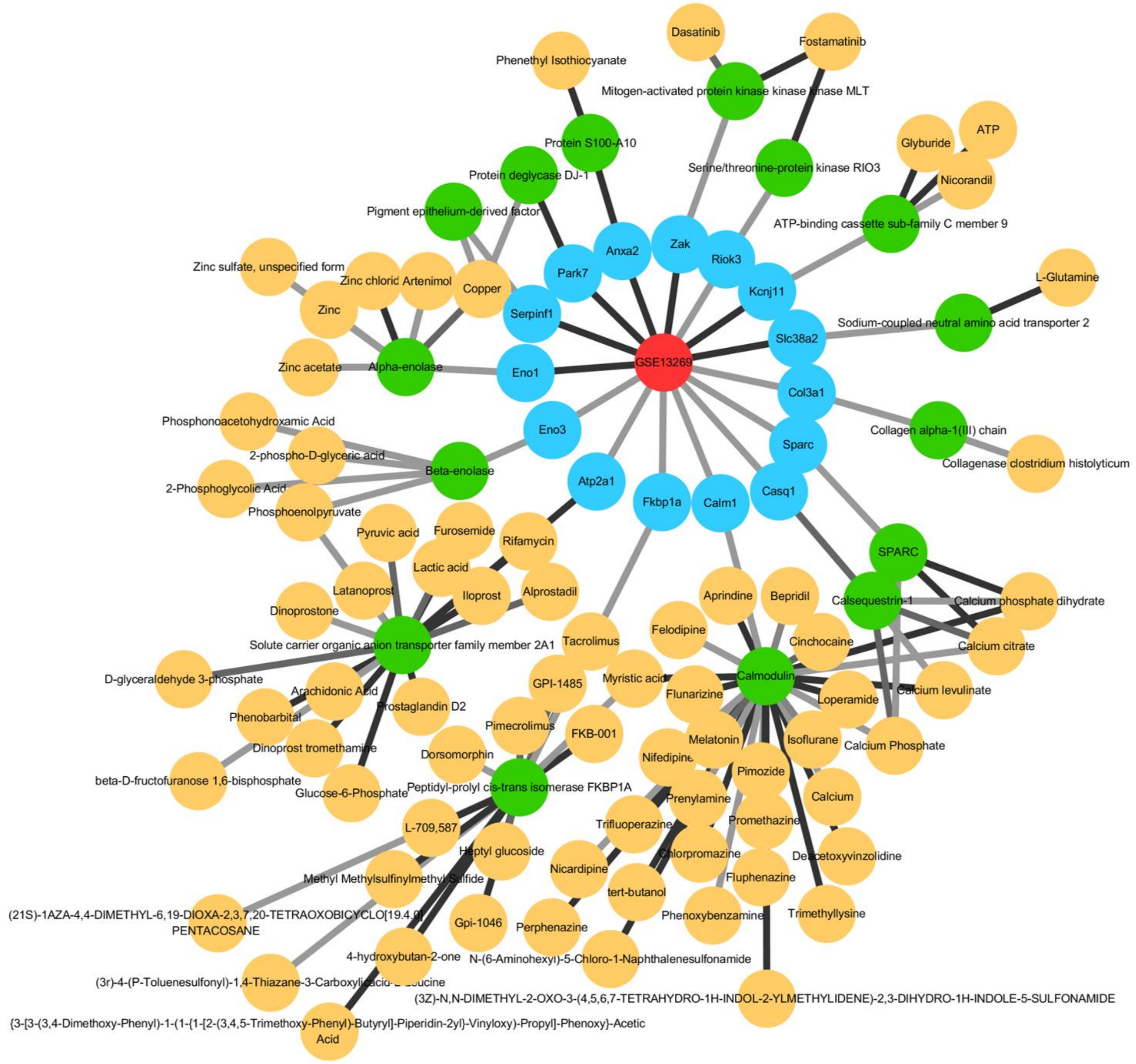

e

Figure S4: The drug target analysis for GSE13269, involving rat gastrocnemius muscle tissue. Drug targets, the corresponding proteins and targeted drugs at (a) stage I; (b) stage II; (c) stage III; (d) stage IV; (e) stage V.

In the first stage of GSE13269, the calsequestrin protein (CASQ) is involved in calcium chelation, storing and releasing calcium within cells, a process that has been shown to mediate the transport of glucose in muscles. CasQ' s gene CASQ1 is encoded in chromosome 1q21, a region that has been shown to be associated with type 2 diabetes in Amish people and several other populations [6]. The single-stranded nucleic acid-binding protein KHSRP (KH-type splicing regulatory protein) regulates the lifespan of RNA and its gene expression at different levels. KHSRP also controls important cellular functions such as proliferation, differentiation, metabolism, and response to infectious agents [7]. In the fourth stage of GSE13269, there is an *in vitro* protein that reduces the process of insulin-stimulated glucose uptake, glycogen incorporation and oxidation by increasing the signaling of the 5'-adenosine monophosphate-activated protein kinase AMPK, while stimulating fatty acid oxidation and incorporation into triglycerides, thereby reducing protein synthesis [8]. We also demonstrated *in vivo* function and effects on overall obesity with the common SERPINF1 variant rs12603825, where the secondary A allele represents plasma PEDF- and the risk of elevated body fat alleles [9].

S5. Drug target analysis for GSE13270: Rat Liver: Rat Liver Tissue

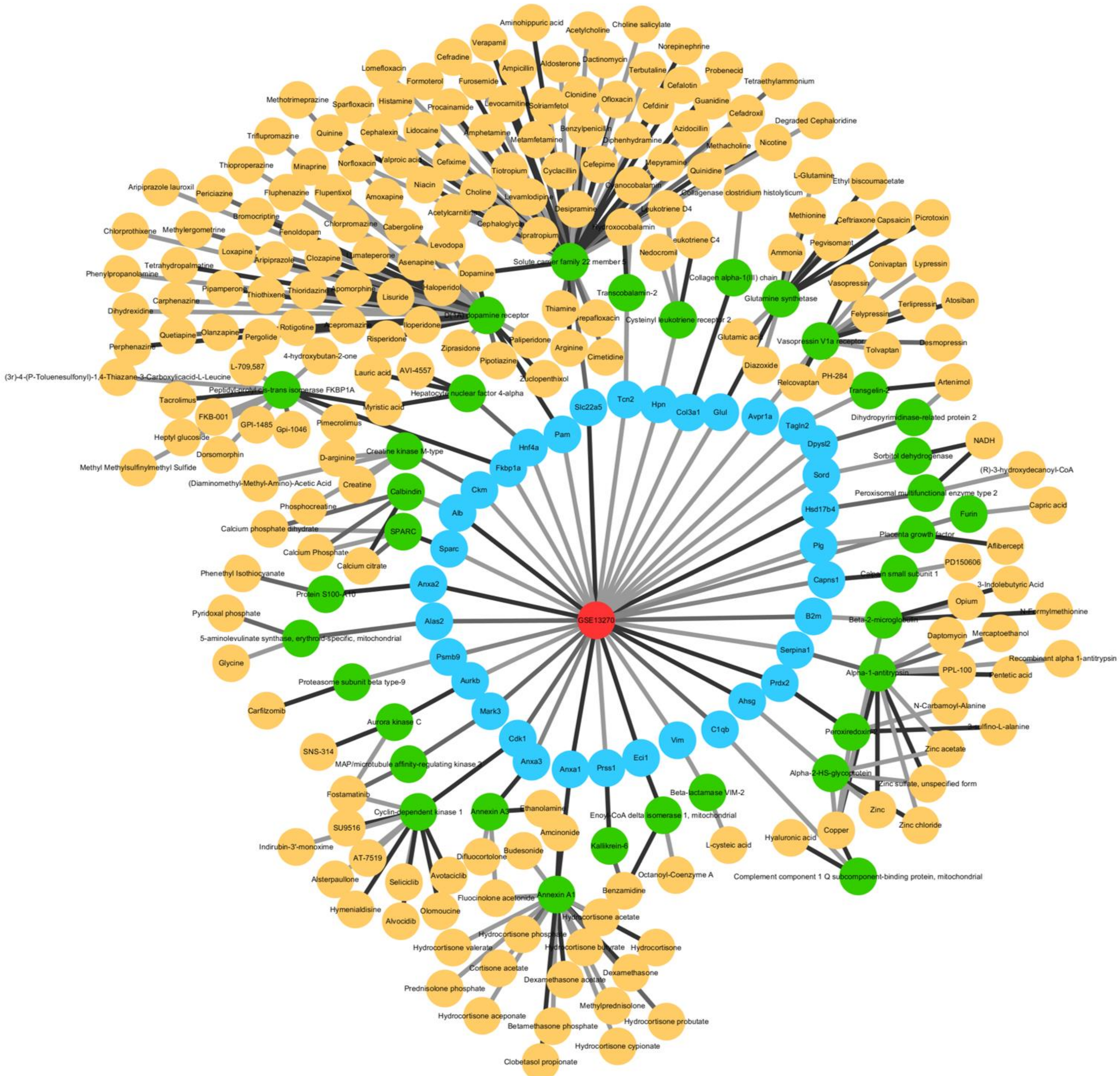

a

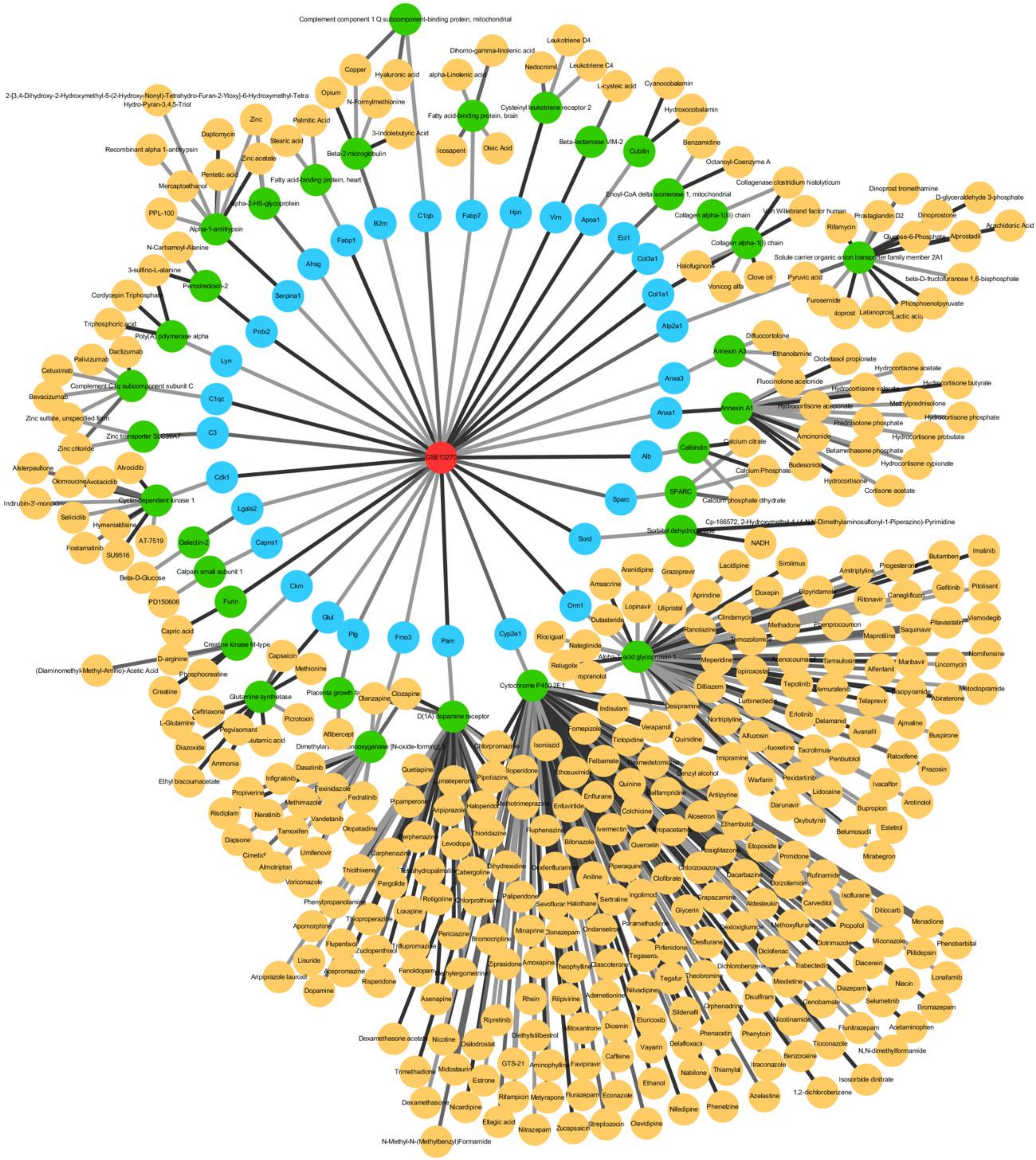

GSE13270
Complement component 1 Q subcomponent-binding protein, mitochondrial
Copper
Hyaluronic acid
Opium
N-Formylmethionine
3-Indolebutyric Acid
Beta-2-microglobulin
Dihomo-gamma-linolenic acid
alpha-Linolenic acid
Fatty acid-binding protein, brain
Icosapent
Oleic Acid
Leukotriene D4
Nedocromil
Leukotriene C4
L-cysteic acid
Cysteinyl leukotriene receptor 2
Cyanocobalamin
Hydroxocobalamin
Beta-lactamase VIM-2
Cubilin
Benzamidine
Octanoyl-Coenzyme A
Collagenase clostridium histolyticum
2-[3,4-Dihydroxy-2-Hydroxymethyl-5-(2-Hydroxy-Nonyl)-Tetrahydro-Furan-2-Yloxy]-6-Hydroxymethyl-Tetra
Hydro-Pyran-3,4,5-Triol
Zinc
Daptomycin
Palmitic Acid
Stearic acid
Recombinant alpha 1-antitrypsin
Zinc acetate
Pentetic acid
Mercaptoethanol
Fatty acid-binding protein, heart
PPL-100
Alpha-1-antitrypsin
N-Carbamoyl-Alanine
3-sulfino-L-alanine
Peroxiredoxin-2
Cordycepin Triphosphate
Triphosphoric acid
Poly(A) polymerase alpha
Daclizumab
Palivizumab
Cetuximab
Bevacizumab
Zinc sulfate, unspecified form
Zinc chloride
Alsterpaullone
Alvocidib
Seliciclib
Hymenialdisine
AT-7519
Fostamatinib
SU9516
Beta-D-Glucose
PD150606
Galectin-2
Calpain small subunit 1
Furin
Capric acid
D-arginine
(Diaminomethyl-Methyl-Amino)-Acetic Acid
Phosphocreatine
Creatine
Glutamine synthetase
L-Glutamine
Ceftriaxone
Pegvisomant
Diazoxide
Ammonia
Ethyl biscoumacetate
Capsaicin
Methionine
Picrotoxin
Aflibercept
Olanzapine
Clozapine
D(1A) dopamine receptor
B2m
C1qb
Fabp7
Hpn
Vim
Apoa1
Eci1
Col3a1
Col1a1
Atp2a1
Fabp1
Ahsg
Serpina1
Prdx2
Lyn
C1qc
C3
Cdk1
Lgals2
Capns1
Ckm
Glul
Plg
Fmo3
Pam
Cyp2e1
Orm1
Sord
Sparc
Alb
Anxa1
Anxa3
Collagen alpha-1(III) chain
Collagen alpha-1(I) chain
Halofuginone
Vonicog alfa
Clove oil
Pyruvic acid
Solute carrier organic anion transporter family member 2A1
Dinoprost tromethamine
D-glyceraldehyde 3-phosphate
Prostaglandin D2
Dinoprostone
Rifamycin
Alprostadil
Arachidonic Acid
beta-D-fructofuranose 1,6-bisphosphate
Phosphoenolpyruvate
Furosemide
Iloprost
Latanoprost
Lactic acid
Annexin A3
Annexin A1
Diflucortolone
Ethanolamine
Clobetasol propionate
Fluocinolone acetonide
Hydrocortisone acetate
Hydrocortisone butyrate
Hydrocortisone aceponate
Methylprednisolone
Hydrocortisone phosphate
Prednisolone phosphate
Hydrocortisone probutate
Amcinonide
Betamethasone phosphate
Hydrocortisone cypionate
Budesonide
Hydrocortisone
Cortisone acetate
Calbindin
Calcium citrate
Calcium Phosphate
SPARC
Calcium phosphate dihydrate
NADH
Aranidipine
Grazoprevir
Lacidipine
Sirolimus
Amsacrine
Lopinavir
Ulipristal
Aprindine
Doxepin
Amitriptyline
Progesterone
Butamben
Imatinib
Gefitinib
Pitolisant
Ritonavir
Methadone
Phenprocoumon
Maprotiline
Saquinavir
Pitavastatin
Vismodegib
Nomifensine
Lincomycin
Alfentanil
Maribavir
Metoclopramide
Abiraterone
Tepotinib
Telaprevir
Erlotinib
Delamanid
Avanafil
Ajmaline
Buspirone
Raloxifene
Prazosin
Ivacaftor
Arotinolol
Bupropion
Estetrol
Mirabegron
Riociguat
Nateglinide
Relugolix
Meperidine
Diltiazem
Desipramine
Nortriptyline
Alfuzosin
Penbutolol
Indisulam
Verapamil
Fomepizole
Ticlopidine
Quinidine
Imipramine
Warfarin
Pexidartinib
Lidocaine
Darunavir
Oxybutynin
Belumosudil
Benzyl alcohol
Antipyrine
Alosetron
Ethambutol
Etoposide
Primidone
Rufinamide
Quetiapine
Pipotiazine
Iloperidone
Isoniazid
Felbamate
Quinine
Enflurane
Colchicine
Ivermectin
Quercetin
Dacarbazine
Dorzolamide
Carvedilol
Isoflurane
Ditiocarb
Menadione
Phenobarbital
Dasatinib
Infigratinib
Propiverine
Fexinidazole
Fedratinib
Risdiplam
Neratinib
Vandetanib
Olopatadine
Tamoxifen
Dapsone
Umifenovir
Almotriptan
Voriconazole
Pipamperone
Aripiprazole
Perphenazine
Levodopa
Thioridazine
Fluphenazine
Bifonazole
Piperaquine
Clofibrate
Aniline
Sertraline
Glycerin
Aldesleukin
Propofol
Miconazole
Plitidepsin
Thiothixene
Pergolide
Cabergoline
Dihydrexidine
Paliperidone
Halothane
Desflurane
Diclofenac
Trabectedin
Diacerein
Niacin
Lonafarnib
Phenylpropanolamine
Rotigotine
Loxapine
Periciazine
Bromocriptine
Minaprine
Ondansetron
Pirfenidone
Dichlorobenzene
Mexiletine
Diazepam
Selumetinib
Bromazepam
Apomorphine
Flupentixol
Zuclopenthixol
Lisuride
Acepromazine
Risperidone
Aripiprazole lauroxil
Dopamine
Fenoldopam
Ziprasidone
Amoxapine
Clascoterone
Tegafur
Theobromine
Disulfiram
Cenobamate
Acetaminophen
Flunitrazepam
N,N-dimethylformamide
Asenapine
Rhein
Rilpivirine
Ademetionine
Sildenafil
Orphenadrine
Nicotinamide
Ripretinib
Diethylstilbestrol
Mitoxantrone
Diosmin
Etoricoxib
Phenacetin
Phenytoin
Tioconazole
Dexamethasone acetate
Nicotine
Osilodrostat
Caffeine
Vayarin
Benzocaine
Isosorbide dinitrate
Trimethadione
Midostaurin
GTS-21
Favipiravir
Ethanol
Nabilone
Thiamylal
Itraconazole
Estrone
Azelastine
1,2-dichlorobenzene
Dexamethasone
Nicardipine
Rifampicin
Metyrapone
Flurazepam
Econazole
Nifedipine
Phenelzine
Ellagic acid
Nitrazepam
Zucapsaicin
Streptozocin
Clevidipine
N-Methyl-N-(Methylbenzyl)Formamide

b

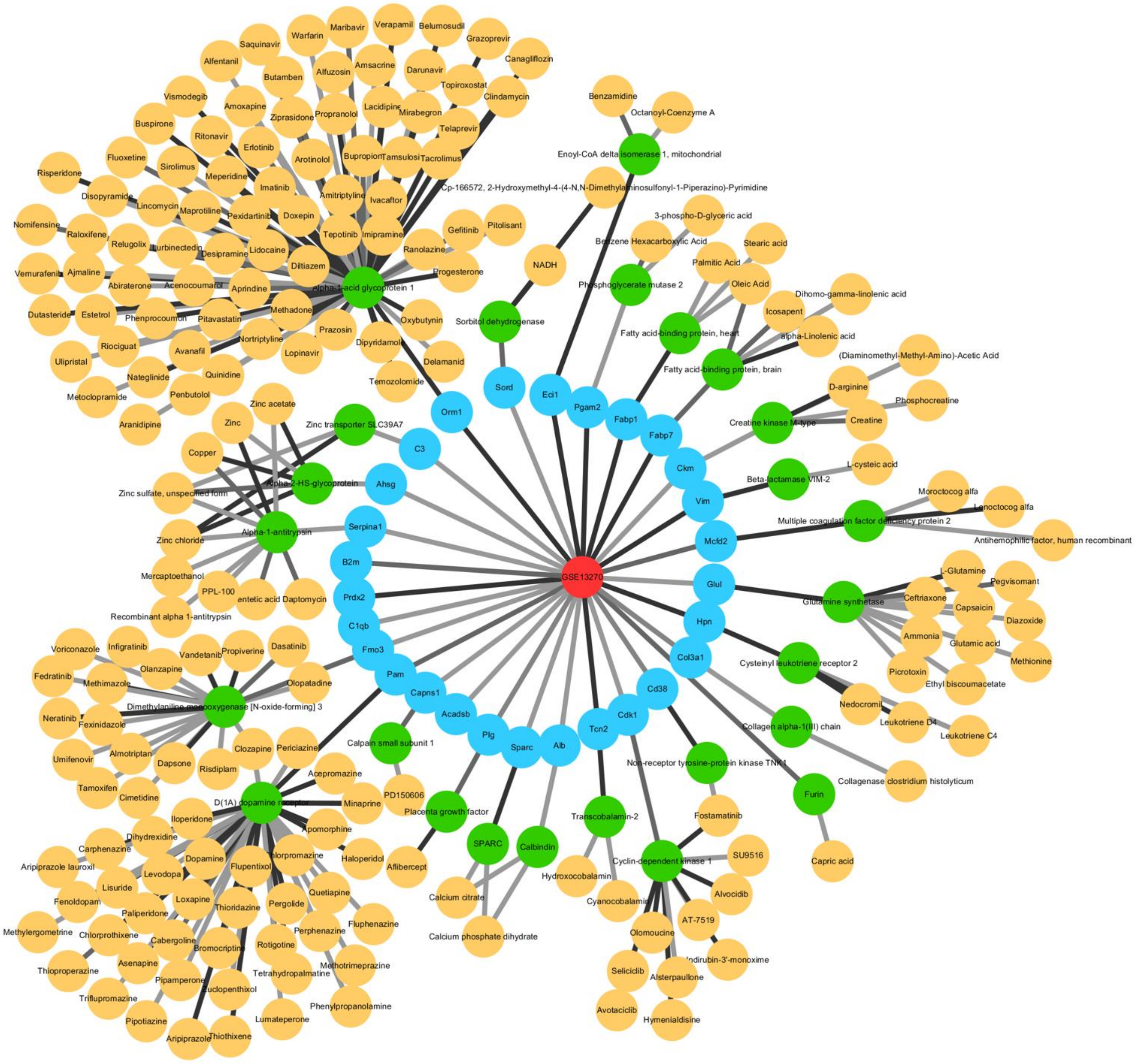

c

Figure S5: The drug target analysis for GSE13270, involving rat liver tissue. Drug targets, the corresponding proteins and targeted drugs at (a) stage II; (b) stage III; (c) stage V.

In addition, the stage I and the stage IV are critical stages, and the corresponding drug target map is put into the body of the paper and analyzed, so it will not be repeated here. The non-critical phase does not have to be analyzed.

S6. Enrichment Results and Analysis of GSE13269 (Rat Gastrocnemius Muscle Tissue) and GSE13270 (Rat Liver Tissue).

For GK rat muscle, the biological processes related in 4 weeks mainly include muscle system process, response to stimulus, response to glucose, heart process, cellular response to glucose stimulus and PI3K-Akt signaling pathway, which are provided in Table S7 and Figure S6 (a). The biological processes in 16 weeks mainly include regulation of peptidase activity, negative regulation of phosphorylation, regulation of necrotic cell death, carbohydrate metabolic process, glycolysis/ gluconeogenesis, HIF-1 signaling pathway, PI3K-Akt signaling pathway, proteoglycans in cancer and response to lipid, which are also provided in Table S7 and Figure S6 (a). For PI3K-Akt signaling pathway, the gene Sirt1 participates in diabetic myocardial injury and the occurrence and development of early diabetic cardemonopathy by negatively regulating PI3K/Akt/MTOR signaling pathway [10]. Previous studies through cell transfection and other experiments have shown that HIF-1α/KIM1 signaling pathway can participate in the process of renal fibrosis in diabetic nephropathy by regulating the expression of KIM1 in renal tubular epithelial cells in high glucose environment. HIF-1α is the key transcription factor of oxygen homeostasis regulation, which can make the body adapt to the external environment oxygen by regulating the expression of target genes. The expression levels of HIF-1α, KIM1, COL-1mRNA and protein in kidney of diabetic rats are significantly increased, suggesting that diabetic nephropathy model is accompanied by changes of HIF-1α/KIM1 pathway in kidney [11]. For some genes in DNB, PRDX2 has been found to participate in the oxidative stress process through a variety of signaling pathways. The hypoxia environment caused by it leads to the increase of reactive oxygen species. In order to survive in a high level of reactive oxygen species, cells need to increase antioxidants, so the expression of related factors, including PRDX2 protein, has changed. Previous studies suggest that overexpression of PRDX2 can protect pancreatic cell apoptosis induced by oxidative stress and re-duce the risk of diabetes [12]. Some studies [13,14] believe that SPARC is an autocrine and/or paracrine factor of adipose tissue, which can inhibit the fat formation. Other studies [15] show that insulin can increase the expression of SPARC, while SPARC can increase the phosphorylation of AKT and PI3-K, indicating that SPARC may be in-volved in insulin signaling pathway [16].

For GK rat liver, the biological processes in 4 weeks mainly include response to hormone, inflammatory response, response to interleukin-1, metabolism of xenobiotics by cytochrome P450, AGE-RAGE signaling pathway in diabetic complications and protein digestion and absorption, which are provided in Table S7 and Figure S6 (b). The biological processes in 16 weeks mainly include liver de-elopement, regulation of secretion, cellular lipid metabolic process, vitamin A metabolic process, regulation of cellular protein metabolic process, PPAR signaling pathway and AGE-RAGE signaling pathway in diabetic complications, which are also provided in Table S7 and Figure S6 (b). Vitamin A is required for PSC (a lipid storage cell, which is normally in a resting state) to maintain a resting state. Vitamin A and its metabolites can inhibit the expression of resting and activated pancreatic stellate cells α-SMA [17]. The peroxisome proliferator-activated receptors (PPARs) modulate several biological processes that are perturbed in obesity, including inflammation, lipid and glucose metabolism and overall energy homeostasis. And PPARs regulate the functions of adipose tissues, such as adipogenesis, lipid storage and adaptive thermogenesis [18]. For the AGE-RAGE signaling pathway, moderate intensity aerobic exercise inhibit-its AGE—RAGE axis and NF-κB pathway, which may decrease oxidative stress and in-Flammarion, and so reduce tissue injure for the prevention and treatment of complications of the T2DM [19]. For some genes in DNB, CD74 receptor and other

mechanisms are stimulated by multi effect cytokine MIF to promote inflammatory response in glomerular podocytes. MIF can activate CD74 on the surface of local glomerular podocytes, resulting in the phosphorylation of extracellular signal regulated kinase 1/2 (ERK1/2) and p38MAPK [20]. SPARC is an autocrine and paracrine factor of adipose tissue, which can inhibit adipogenesis. Insulin can increase SPARC expression, while SPARC can in-crease Akt and P13-K phosphorylation, indicating that SPARC may be involved in insulin signaling pathway [21]. The overexpression of PRDX2 can protect pancreatic β cell apoptosis induced by oxidative stress and reduce the risk of diabetes. The changes of oxidative stress in diabetic kidney disease and other pathological processes are related to the expression of PRDX2 and related pathways. For example, PRDX2 can participate in the regulation of tumor development and oxidative stress in treatment through P13 AKT-resistant pathway. PRDX2 can regulate the oxidative stress of colon cancer cells through Wnt/B-catenin signaling pathway and influence the oxidative stress of tumor by regulating some microRNA [22].

Table S7. Enrichment results for DNBs of GSE13269 (rat gastrocnemius muscle tissue) and GSE13270 (rat liver tissue) datasets.

Tissue	Case	Term	P-value	Term name
Muscle	Muscle 4 weeks	GO:0003012	1.05×10^{-5}	Muscle system process
		GO:0050896	0.0067	Response to stimulus
		GO:0009749	0.0121	Response to glucose
		GO:0003015	0.0334	Heart process
		GO:0071333	0.0351	Cellular response to glucose stimulus
		GO:0042593	0.0458	Glucose homeostasis
		rno04260	3.72×10^{-8}	Cardiac muscle contraction
		rno00190	1.95×10^{-7}	PI3K-Akt signaling pathway
	Muscle 16 weeks	GO:0052547	0.0048	Regulation of peptidase activity
		GO:0042326	0.0117	Negative regulation of phosphorylation
		GO:0010939	0.0176	Regulation of necrotic cell death
		GO:0005975	0.0239	Carbohydrate metabolic process
		rno00010	0.00045	Glycolysis / Gluconeogenesis
		rno04066	0.0075	HIF-1 signaling pathway
		rno04151	0.0092	PI3K-Akt signaling pathway
		rno05205	0.0213	Proteoglycans in cancer
Liver	Liver 4 weeks	GO:0033993	1.63×10^{-11}	Response to lipid
		GO:0009725	5.36×10^{-9}	Response to hormone
		GO:0006954	0.0002	Inflammatory response

	GO:0002443	0.00033	Leukocyte mediated immunity
	GO:0070555	0.0292	Response to interleukin-1
	rno00980	0.0172	Metabolism of xenobiotics by cytochrome P450
	rno04933	0.0369	AGE-RAGE signaling pathway in diabetic complications
	rno04974	0.0438	Protein digestion and absorption
Liver 16 weeks	GO:0001889	0.00075	Liver development
	GO:0051046	0.0056	Regulation of secretion
	GO:0044255	0.0208	Cellular lipid metabolic process
	GO:0006776	0.0336	Vitamin a metabolic process
	GO:0032268	0.0473	Regulation of cellular protein metabolic process
	rno03320	0.0039	PPAR signaling pathway
	rno04933	0.0077	AGE-RAGE signaling pathway in diabetic complications
	rno00982	0.0098	Drug metabolism - cytochrome P450

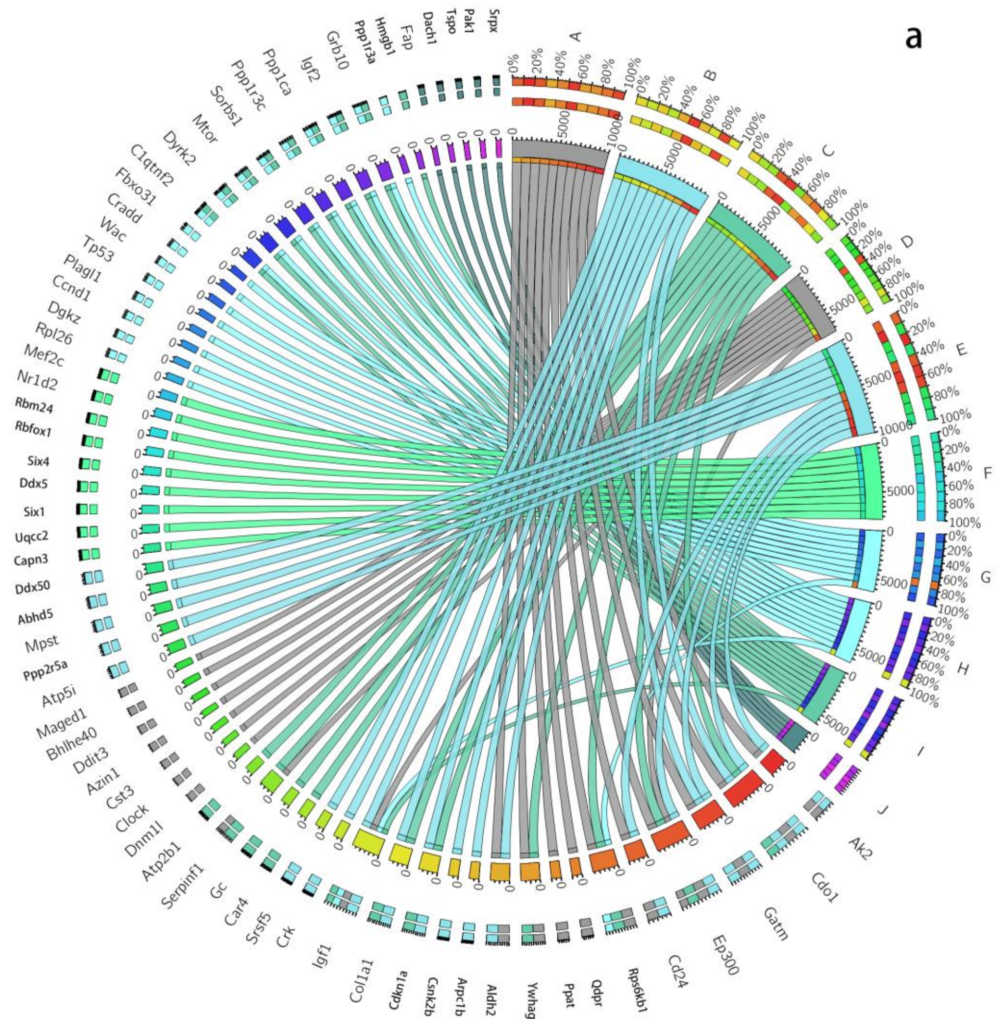

A. Response to hormone

B. Response to peptide hormone

C. Response to steroid hormone

D. Rhythmic process

E. Metabolic process

F. Regulation of skeletal muscle cell differentiation

G. G1 DNA damage checkpoint

H. Regulation of glycogen biosynthetic process

I. Regulation of glycogen metabolic process

J. Contact inhibition

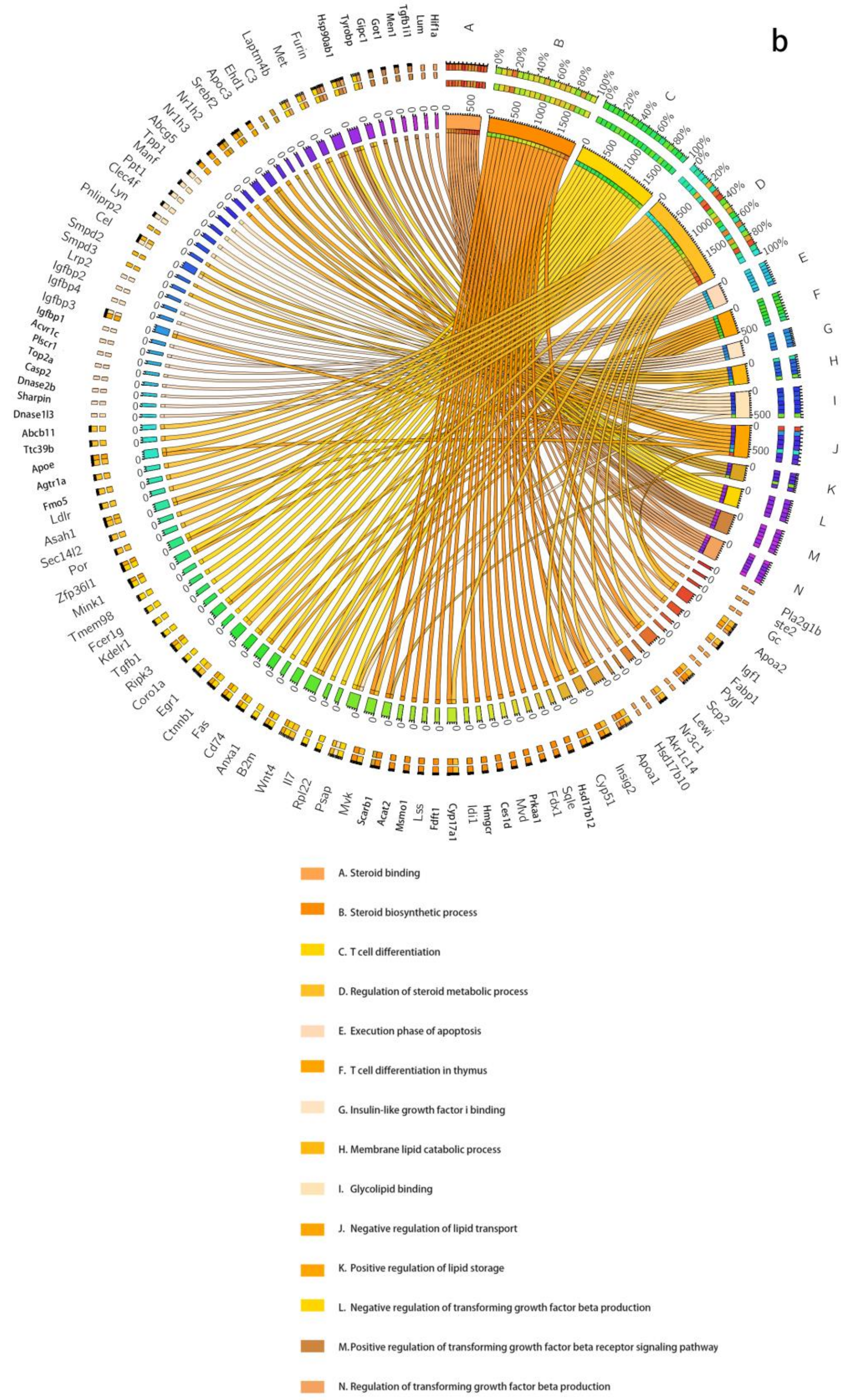

Figure S6. The DNBs of GSE13269, involving rat muscle tissue, and GSE13270, involving rat liver tissue, are involved in important biological processes of T2DM. (a) GSE13269. (b) GSE13270.

References

[1] Dai, H.; Li, L.; Zeng, T.; Chen, L. Cell-specific network constructed by single-cell RNA sequencing data. Nucleic Acids Res. 2019, 11.

[2] Johnson J.M.; Edwards, S.; Shoemaker, D.; Schadt E.E. Dark Matter in the Genome: Evidence of Widespread Transcription Detected by Microarray Tiling Experiments. Trends Genet 2005, 21, 93–102.

[3] Lin, G.; Wan, X.; Liu, D.; Wen, Y.; Yang, C; Zhao, C. COL1A1 as a potential new biomarker and therapeutic target for type 2 diabetes. Pharmacol Res. 2021, 165, 105436.

[4] Wang, X.; Lu, J. Effect of blood glucose fluctuation on the prognosis of diabetes and the occurrence and development of chronic complications. Section Endocrinol Foreign M ed Sci 2005, 25.

[5] Hoshino, F.; Murakami, C.; Sakai, H.; Satoh, M.; Sakane, F. Creatine kinase muscle type specifically interacts with saturated fatty acid-and/or monounsaturated fatty acid-containing phosphatidic acids. Biochem. Biophys. Res. Commun. 2019, 513, 1035-1040.

[6] Fu, M.; Damcott, C.M.; Sabra, M.; Pollin, T.I.; Ott, S.H.; Wang, J; Garant, M.J.; O'connell, J.R.; Mitchell, B.D.; Shuldiner, A.R. Polymorphism in the Calsequestrin 1 (*CASQ1*) Gene on Chromosome 1q21 Is Associated with Type 2 Diabetes in the Old Order Amish. Diabetes 2004, 53, 3292-3299.

[7] Briata, P.; Bordo, D.; Puppo, M.; Gorlero, F.; Rossi, M.; Perrone-Bizzozero, N.; Gherzi, R. Diverse roles of the nucleic acid-binding protein KHSRP in cell differentiation and disease. Wiley Interdiscip Rev RNA 2016, 7, 227-240.

[8] Ruby, M.A.; Riedl, I.; Massart, J.; M Åhlin.; Zierath, J.R. Protein kinase N2 regulates AMP kinase signaling and insulin responsiveness of glucose metabolism in skeletal muscle. Am J Physiol Endocrinol Metab. 2017, 313, 483-491.

[9] Anja, B.; Anna-Maria, O.; M Jürgen.; Mratin, H.; Caroline, K.; Fausto, M.; Fritz, S.; Norbert, S.; Andreas, F.; Hans-Ulrich, H. Common genetic variation in the SERPINF1 locus determines overall adiposity, obesity-related insulin resistance, and circulating leptin levels. 2012, 7, e34035.

[10] Sun, X.H.; Wang, Y.; Mou, Y. Alteration of PI3K/Akt/mTOR signaling during development of diabetic cardiomyopathy and regulation of SIRT1. Chinese Pharmacol. Bull. 2017, 33, 793-798.

[11] Liu, J.; Hu, C.; Li, H.; Wang, W.; Zhang, W.; Niu, C.; Chen, M. Based on Kim1 / HIF - 1 α Signaling pathways explore the mechanism of kidney injury in diabetic rats. Chinese Journal of Integrated Traditional and Western Nephrology 2020, 21, 786-788.

[12] Yao, J.; Jiang, A.; Wang, L.; Wei, F.; Chen, H.; Yu, H.; Meng, J. Triptolide upregulated PRDX2 inhibits oxidative stress in type 2 diabetic nephropathy. Journal of Tianjin Medical University 2019, 25, 466-470.

[13] Jing Nie. E; Helence Sage. SPARC functions as all inhibitor of adipogenesis . J Cell Commun Sigmal 2009,3,247-254 .

[14] Kos, K.; Wong S.; Tan B. et al. Regulation of the fibrosis and angiogenesis

promoter sparc/osteonectin in human adipose tissue by weight change, leptin, insulin, and glucose . Diabetes 2009, 58, 1780-1788 .

[15] Shi, Q.; Bao S.; Maxwell J.A.; et a1 . Secreted protein acidic, rich in eysteine (SPARC), mediates cellular survival of gliomas through AKT activation . J Biol Chem 2004, 279,52200—52209 .

[16] Jia, G.; Di, F.; Wang, L.; Yu, H.; Li, X. Association between the serum SPARC and FGF21 levels in newly diagnosed T2DM patients. China Diabetes journal 2013, 21, 223-225.

[17] She, Y.; Gong, W.; Yu, J. Effect of Vitamin A on Islet Fibrois in Type 2 Diabetes Mellitus Animal Model. Laboratory Animal Science and Administration 2015, 32, 17-20.

[18] Gross, B.; Pawlak, M.; Lefebvre, P.; Staels, B. PPARs in obesity-induced T2DM, dyslipidaemia and NAFLD. Nature reviews. Endocrinology 2017, 13, 36-49.

[19] Shen, Z.; Zhu, L.; Xu, W. Effects of aerobic exercise on AGE-RAGE axis and NF-κB pathway in type 2 diabetic rats. Kunming Med. Univ. 2018, 39, 16-19.

[20] Liu, J.; Ye, S.; Bi, S.; Zhou, W. Effect of metformin on the expression of macrophage migration inhibitory factor and its receptor CD74 in renal tissue of type 2 diabetic rats. Acta Universitatis Medicinalis Anhui 2021, 56, 5.

[21] Jia, G.; Di, F.; Wang, L.; Yu, H.; Li, X. Correlation between serum SPARC and FGF21 levels in patients with new type 2 diabetes mellitus. Chin J Diabetes 2013, 21, 5.

[22] Yao, J.; Jiang, A.; Wang, L.; Wei, F.; Chen, H.; Yu, H.; Meng, J. Triptolide upregulates PRDX2 and inhibits oxidative stress in type 2 diabetic nephropathy. Tianjin Med. Univ. 2019, 25, 5.
